# Immune pathogenesis in pigeons during experimental *Prohemistomum vivax* infection

**DOI:** 10.3389/fvets.2022.974698

**Published:** 2022-09-14

**Authors:** Asmaa M. I. Abuzeid, Mahmoud M. Hefni, Yue Huang, Long He, Tingting Zhuang, Guoqing Li

**Affiliations:** ^1^Guangdong Provincial Key Laboratory of Zoonosis Prevention and Control, College of Veterinary Medicine, South China Agricultural University, Guangzhou, China; ^2^Department of Parasitology, Faculty of Veterinary Medicine, Suez Canal University, Ismailia, Egypt; ^3^Institute of Biotechnology for Postgraduates Studies and Researches, Suez Canal University, Ismailia, Egypt

**Keywords:** *Prohemistomum vivax*, trematode, pigeons, phylogeny, RT-qPCR, cytokines, gene expression, immune pathogenesis

## Abstract

*Prohemistomum vivax* is a small trematode belonging to the family Cyathocotylidae, infecting fish-eating birds and mammals, including humans. However, no data on molecular identification and immune pathogenesis are available, challenging effective diagnostic and therapeutic interventions. Here, we identified *P. vivax* based on combined morphological and molecular data and examined histopathological lesions and the differential cytokines expression in experimentally infected pigeons. Pigeons were orally infected with 500 prohemistomid metacercariae. Intestinal and spleen tissues were harvested 2, 4, 7, 14, 21, and 28 days post-infection (dpi). Gene expression levels of eleven cytokines (IL-1, IL-2, IL-4, IL-5, IL-6, IL-10, IL-12, IL-15, IL-18, IFN-γ, and TGF-β3) were assessed using quantitative reverse-transcription PCR (RT-qPCR). We identified the recovered flukes as *Prohemistomum vivax* based on morphological features and the sequence and phylogenetic analysis of the internal transcribed spacer 1 (ITS1), 5.8 ribosomal RNA, and ITS2 region. Histopathological lesions were induced as early as 2 dpi, with the intensity of villi atrophy and inflammatory cell infiltration increasing as the infection progressed. An early immunosuppressive state (2 and 4 dpi), with TGF-β3 overexpression, developed to allow parasite colonization. A mixed Th1/Th2 immune response (overexpressed IFN-γ, IL-12, IL-2, IL-4, and IL-5) was activated as the infection progressed from 7 to 28 dpi. Inflammatory cytokines (IL-1, IL-6, IL-18, and IL-15) were generally overexpressed at 7–28 dpi, peaking at 7 or 14 dpi. The upregulated Treg IL-10 expression peaking between 21 and 28 dpi might promote the Th1/Th2 balance and immune homeostasis to protect the host from excessive tissue pathology and inflammation. The intestine and spleen expressed a significantly different relative quantity of cytokines throughout the infection. To conclude, our results presented distinct cytokine alteration throughout *P. vivax* infection in pigeons, which may aid in understanding the immune pathogenesis and host defense mechanism against this infection.

## Introduction

Cyathocotylidae Mühling, 1896 is a small, globally distributed digenean trematode family (superfamily Diplostomoidea Poirier, 1886), infecting birds, mammals, and reptiles (1). The taxonomy of this family has been controversial. According to most recent reports, this family is divided into five subfamilies, Cyathocotylinae Mühling, 1898; Prohemistominae Lutz, 1935; Szidatiinae Dubois, 1938; Prosostephaninae Szidat, 1936; and Muhlinginae Mehra, 1950. *Prohemistomum vivax* (*P. vivax*) belongs to the subfamily Prohemistominae which includes five genera: *Prohemistomum* Odhner, 1913; *Mesostephanus* Lutz, 1935; *Mesostephanoides* Dubois, 1951; *Paracoenogonimus* Katsurada, 1914; and *Linstowiella* Szidat, 1933 (1). *Prohemistomum vivax* inhabits the intestine of fish-eating birds and mammals, including humans, and has been recorded in Egypt, Israel (Palestine), Japan, and Europe (2, 3). This intestinal fluke can be transmitted by consuming infected fish intermediate hosts. *Prohemistomum vivax* intermediate hosts include a variety of fresh and brackish water fish, such as *Tilapia zilli, Tilapia nilotica, Clarias gariepinus, Clarias lazera, Chrysichthys auratus, Bagrus bayad, Barbus binny, Ctenopharyngodon idella, Gambusia affinis, Shilbe mystus, Hydrocyon* sp., *Atherina* sp., *Alestes* sp., *Eutropius* sp., *Schilbe* sp., *Mugil capito*, and *Mugil cephalus* (2, 4–7). Fish with metacercarial infection display myositis, muscle pressure atrophy, respiratory distress, excessive mucus, scale loss, and spots on affected tissues, with significant economic losses (7, 8). The zoonotic and economic impact of *P. vivax* necessitates developing an effective tool for diagnosis and control.

The current systematics of the family Cyathocotylidae mainly depend on morphological features. Molecular data and phylogenetic studies of this family are lacking. Globally, DNA sequences are available from adults of only four species from reptile hosts belonging to genera *Suchocyathocotyle* and *Gogatea* (9) and eight species from avian hosts belonging to genera *Holostephanus, Holostephanoides, Cyathocotyle, Mesostephanus*, and *Neogogatea* (9–13). Several cyathocotylid trematodes were recorded in Egypt, including *Prohemistomum vivax, Prohemistomum azimi* n. sp., *Mesostephanus appendiculatus, Mesostephanus burmanicus, Mesostephanus milvi, Mesostephanus odhneri*, and *M. fajardensis* (4, 14, 15). However, these species are morphologically similar, and molecular data were recovered from adults of only one species, *M. appendiculatus* (13). Thus, more efforts are needed to develop a fast and accurate molecular tool to differentiate these trematodes.

Intestinal trematode infections can cause significant pathological alterations in the gut of final hosts, leading to enteritis (16). *Prohemistomum vivax* in experimentally infected rats resulted in intestinal villi ulceration and deformation, shortening, blunting, and fusion. In the intestinal lamina propria, crypt hypertrophy with inflammatory cell infiltration was also observed (17). Although Amer (18) reported mast cells and eosinophils efflux in the intestinal mucosa of infected rats, intestinal mastocytosis plays a minor role in intestinal trematode expulsion (19). Therefore, more research is essential to clarify the immune mechanisms and effecter cells involved in host damage and worm expulsion during *P. vivax* infection.

In mammals, T-helper (Th) cell immunity, either Th1 or Th2, or both, is activated in response to helminth parasites. Th2 response is characterized by the production of interleukin (IL)-4, IL-5, IL-9, and IL-13. These interleukins trigger mast cells and eosinophils and raise IgE and IgG1 serum levels (20). Nevertheless, Th1 cells produce interferon (IFN)-γ and are linked to the release of proinflammatory cytokines, inhibiting Th2 responses. A successful resolution of infection requires balanced Th1 and Th2 responses, while an unbalanced response causes host damage (21). Like their mammalian counterparts, avian cytokines play a role in the host immune response to pathogenic infection. *Ascaridia galli*-infected pigeons had significantly higher IL1-β and tumor necrosis factor (TNF-α) levels than apparently healthy pigeons (22). IFN-γ and TNF-α cytokines were significantly upregulated in domestic pigeons during the late central-nervous phase of Apicomplexa protozoon parasite (*Sarcocystis calchasi*) infection (23). Nevertheless, little is known about cytokines' role during trematode infection in pigeons. Generally, the selective stimulation of Th1 and Th2 cells in helminthic infections differs depending on the parasite species. On the one hand, the host protection and worm expulsion during intestinal nematode infections mainly rely on the Th2 response (24). On the other hand, little is known about host Th responses in intestinal trematode infections, except for *Echinostoma caproni* and *Neodiplostomum seoulense* infections (25–27). Immune responses were biased toward a Th1 phenotype in *E. caproni* (25), while mixed Th1 and Th2 responses were activated during *N. seoulense* infection (26). However, no studies have investigated Th immune responses and related cytokines during infection with members of the Cyathocotylidae family. This study aims to identify *P. vivax* trematode based on morphology and internal transcribed spacer sequence and to evaluate the effect of this fluke on cytokines gene expression in the intestine and spleen of experimentally infected pigeons.

## Materials and methods

### Parasite isolation

Encysted Metacercariae (EMC) were collected from infected African catfish (*Clarias gariepinus*), purchased from fish markets in Ismailia city, Ismailia Province, Egypt (30° 35′ 0″ N, 32° 16′ 0″ E). Minced muscle samples were exposed to the acid-pepsin solution (10 g 1:3000 pepsin powder (Oxford Lab Fine Chem LLP, Navghar, India), 10 mL of 25% HCl, and 2,000 mL distilled H_2_O; pH 2) and incubated at 37°C for 2–3 h with frequent stirring (28). After filtering the suspensions through a tea sieve, the filtrate was rinsed in 0.85% saline and examined under a stereomicroscope for metacercariae. The prohemistomid metacercariae were concentrated by sedimentation and morphologically identified according to Patarwut et al. (29). Metacercariae were counted and kept in 0.85% saline for <6 h before being used in the experimental infection on the same day.

### Experimental infection

Three to 4-week-old healthy domestic pigeons (*Columba livia domestica*) were purchased from a local breeder. Birds were confirmed as parasite-free by fecal examination repeated for three successive days. In a preliminary experiment, two groups of pigeons (three pigeons/group) were infected with 1,000 or 2,000 EMC/bird. All birds died within a week. Therefore, we used 500 EMC/ bird as the infection dose in the further experiment. In this experiment, pigeons were divided into seven groups. Each group was housed in a well-ventilated cage and received fresh water and feed *ad libitum* throughout the study. Pigeons in experimental groups 1–6 (five pigeons per group) were fed on 500 EMC using a Pasteur pipette. Fecal samples from infected groups were examined daily. The negative control (NC) group (*n* = 12, two for each time point) remained uninfected.

### Gross examination and worm recovery analysis

We euthanized uninfected and infected pigeons at six-time points, 2-, 4-, 7-, 14-, 21-, and 28-days post-infection (dpi), and harvested intestines and spleens. Spleen tissue samples and a portion of the small intestine (contained flukes following washing of ingesta with 0.85% saline) were collected in sterile Eppendorf tubes, snap-frozen in liquid nitrogen, and stored at −80°C. The remaining intestine was assessed for the presence of pathological lesions and flukes. After repeated washing and sedimentation of intestinal contents, we counted the number of worms recovered from each bird under a stereomicroscope.

### Morphometric analysis of parasites

Adult parasites were fixed in AFA (85 mL of 85% ethanol, 10 mL of formalin, and 5 mL of glacial acetic acid) for 10 min and then rinsed in 70% Ethanol for 3 min. Samples were stained in Semichon's acetocarmine stain for 5 min, washed twice in 70% Ethanol, de-stained in 1% acid alcohol for 5 s, and rinsed twice in alkaline alcohol for 3 min. Following dehydration in graded ethanol concentration, 95% (15 min) and 100% (twice, 15 min each), samples were cleared with xylene for 1 min and mounted on microscope slides in Canada balsam. Parasites were photographed using a Leica DM1000 microscope (Leica Microsystems, Wetzlar, Germany) at 100 × magnification, and measurements were obtained with the aid of the ImageJ software (LOCI, University of Wisconsin, USA). Our measurement data were presented as the range (mean ± SD). For taxonomic identification, we compared worm morphological features and measurement data to previous studies (3, 30–34).

### Molecular identification of parasites

Genomic DNA was extracted from 10 flukes stored at −20°C using a QIAGEN DNeasy™ tissue kit (Qiagen, Hilden, Germany) following the manufacturer's instructions. The gDNA concentrations were evaluated by a NanoDrop ND-1000 Spectrophotometer (Thermo Fisher Scientific, Waltham, MA, USA) to optimize the amount of gDNA used in PCR reactions. The isolated gDNA was stored at −20°C until further use. To identify adult flukes, we selected the nuclear ribosomal internal transcribed spacer region, including part of internal transcribed spacer 1 (ITS1), 5.8S ribosomal RNA complete sequence, and part of ITS2. We used the universal primers BD1 (5′-GTCGTAACAAGGTTTCGGTA-3′) and BD2 (5′-TATGCTTAAATTCAGCGGGT-3′) (35, 36) synthesized by FASMAC Co. Ltd. (Atsugi, Japan). The PCR reactions (25 μL) comprised 12.5 μL EmeraldAmp MAX Master Mix (Takara Bio, Kusatsu, Japan), 1 μL of each primer (10 pmol/μL), 20 ng of template DNA, and ddH_2_O up to 25 μL. The PCR was run in a SensoQuest Labcycler 48 (SensoQuest GmbH company, Göttingen, Germany). PCR conditions were pre-denaturation at 94°C for 3 min; 30 cycles of denaturation at 94°C for 1 min, annealing at 56°C for 1 min, and extension at 72°C for 2 min; with a final extension at 72°C for 5 min. The amplified PCR products were examined by 1% ethidium bromide-stained agarose gel electrophoresis. After cleaning using the QIAquick PCR Purification Kit (Qiagen, Hilden, Germany), PCR products were sent to Sangon Biotech Co., Ltd (Shanghai, China) for sequencing in both directions.

### Bioinformatic analysis

We determined homologous sequences using the Basic Local Alignment Search Tool (BLAST) on NCBI. Boundaries between ITS1, 5.8S rRNA, and ITS2 sequences were determined by aligning and comparing our sequence to that of *Cyathocotyle prussica* (MH521249). We selected 27 Diplostomata sequences for phylogenetic analysis. Selected fragments of the ITS1-5.8S-ITS2 region were assembled by MEGA X (37) and aligned with the Clustal W software (38). Homology percent and pairwise distances were analyzed using the Megalign module of the DNASTAR Lasergene package (v. 7.1.0). The alignments dataset was analyzed using MEGA X to predict the best-fitting nucleotide substitution model based on the Akaike Information Criterion (AIC). Maximum likelihood (ML) and Neighbor-joining (NJ) analyses were performed under the K2 + G model. Bootstrap values of 1,000 resampled datasets were used to estimate the nodal support of the phylogenetic tree. The tree explorer of MEGA X was utilized to visualize the phylogenetic trees. *Clonorchis sinensis* (MF319655) was used as an outgroup.

### Histopathological examination

Small intestine tissue samples from each group were fixed in 10% formalin overnight, washed with ddH_2_O, dehydrated in ascending ethanol concentrations (30–100%), cleared with xylene, and embedded in paraffin wax. Sections of 3 μm thickness were sliced and dewaxed with xylene before rinsing in descending ethanol grades (100–30%) and ddH_2_O. After staining with hematoxylin and eosin (HE), sections were dehydrated. Slides were scanned, and images were processed using the ImageJ software (LOCI, University of Wisconsin, United States). Intestinal tissue sections were examined for pathological findings and scored for the following: in?ammation with villous atrophy (none = 0, slight = 1, moderate = 2, and severe = 3), in?amed area (mucosa = 1, mucosa and submucosa = 2, and transmural = 3), surface ulceration (none = 0, focal = 1, diffuse= 2, complete loss of surface epithelium = 3, entire surface epithelium and crypt epithelium are lost = 4), and involvement percentage (1–25% = 1, 26–50% = 2, 51–75% = 3, and 76–100% = 4) (39).

### Quantification of cytokine gene expression

Total RNAs were extracted from intestines and spleen samples stored at −80°C using the Total RNA Purification Kit (Jena Bioscience, Jena, Thuringia, Germany) following the manufacturer′s protocol. A 40 μL DEPC water was added to elute the RNA. The eluted RNA was quantified by a NanoDrop ND-1000 Spectrophotometer (Thermo Scientific, Waltham, MA, USA) and examined by 2% agarose gel electrophoresis to evaluate the integrity of RNA. Before the first-strand cDNA synthesis, the retained genomic DNA in RNA samples was removed as follows: RNA sample (1 μg), 10X reaction buffer with MgCl_2_, DNase I (1 μl), and DEPC-treated Water (up to 10 μl) were placed in an RNase-free tube and incubated at 37°C for 30 min. Then, 50 mM EDTA (1 μL) was added, followed by incubation at 65°C for 10 min. The resulted RNA was used as a template to synthesize the first-strand cDNA by reverse-transcription according to the manufacturer's instructions of the RevertAid First-Strand cDNA Synthesis Kit (Thermo Scientific, Waltham, MA, USA). The cDNA was chilled on ice and stored at −20°C.

Gene expression of eleven cytokines (IL-1, IL-2, IL-4, IL-5, IL-6, IL-10, IL-12, IL-15, IL-18, IFN-γ, and TGF-β_3_) in pigeon intestines and spleen at different time points were evaluated by quantitative reverse-transcription polymerase chain reaction (RT-qPCR). We designed IL-2, IL-4, and IL-5 primers based on conserved regions in genes isolated from related birds available on NCBI. Primers for amplifying other cytokines and internal reference (β*-*actin) genes were synthesized based on published sequences (Table 1). The Applied Biosystems™ 7,500 Real-Time PCR Systems (Applied Biosystem, Bedford, MA, USA) was used to evaluate cytokine expression levels in pigeons during *P. vivax* infection. The qPCR system was consisted of 0.5 μL cDNA (750 ng), 0.5 μL of each forward and reverse primers (10 pmol/L), 5 μL WizPure™ qPCR Master (SYBR) (Wizbiosolution, Gyeonggi-do, Republic of Korea), 0.2 μL ROX Dye (50X), and ddH_2_O up to 10 μL. The qPCR reaction conditions were as follows: pre-denaturation at 95°C for 10 min and 40 cycles of denaturation at 95°C for 15 s and annealing at 58°C for 1 min. The product specificity was detected by a melting curve program at 95°C for 15 s, 60°C for 1 min, 95°C for 30 s, and 60°C for 15 s. Each experiment was conducted in triplicates. The relative expression levels of different cytokines were determined with β*-*actin as the reference gene using the 2^−Δ*ΔCT*^ method (40).

**Table 1:** Primers used for evaluating the effect of *P. vivax* infection on cytokines expression in *P. vivax*-infected pigeons by the quantitative real-time PCR.

**Target gene**	**Primer sequence (5^′^-3^′^)**	**Product size (bp)**	**References**
β-actin	F: AGGCTACAGCTTCACCACCAC R: CCATCTCCTGCTCAAAATCCA	95	(41)
IL1	F: CGAGAGCAGCTACGCCG R: GCCGCTCAGCACACACG	271	(23)
IL-2	F: CCAAATGAGACCAAGGAGTGC R: GCATTCACTTCCTGTGGGATTTAG	179	Present study
IL-4	F: AGACATCCACGCTGCTGAAG R: CGTTACTCTTGTCACAGGAAACC	80	Present study
IL-5	F: CTTTGAAGGAACGGAACTGTTG R: AGGTGTGGTGTGAGTGATTGC	101	Present study
IL-6	F: AGCGTCGATTTGCTGTGCT R: GATTCCTGGGTAGCTGGGTCT	107	(41)
IL-10	F: TGATGAACTTAGCATCCAGCTACTC R: AACTGCATCATCTCCGACACA	93	(41)
IL-12	F: AGTGAAGGAGTTCCCAGATGC R: TTCCAGAGTAGTTCTTTGCCTCAC	188	(23)
IL-15	F: GAATGCCAGGAACCTGTAATG R: GCATTCCCTCTGTATAACCTTTAC	246	(23)
IL-18	F: GCCAGTTGCTTGTGGTTCG R: TCTACCTGGACGCTGAATGC	160	(23)
IFN*-γ*	F: CAAGTCAAAGGCGCACGTC R: GCGTTGAGTTTTCAAGTCATTC	136	(41)
TGF*-β*3	F: AGGACCTTGGCTGGAAATG R: ACCGTGCTGTGAGTGGTGT	106	(41)

F, forward primer; R, reverse primer.

### Statistical analysis

We used GraphPad Prism (GraphPad Software Inc., La Jolla, CA, USA, version 8.0.2) for statistical analysis and graph drawing unless otherwise stated. The difference in the worm recovery between infected groups as well as measurements of each organ of the parasite in our study with those of previous studies were compared using the one-way analysis of variance (ANOVA) followed by LSD multiple comparison test in CoStat 6.45. The Student's *T*-test was used to compare cytokine mRNA expression levels in the uninfected and infected groups at various time points after infection. We used Pearson's correlation coefficient (*r*-value) with a two tailed test to measure the pairwise relationship between Th1, Th2, and Treg cytokine expression levels in infected groups. Cytokine relative quantities were compared in the intestine and spleen of infected groups at each time point using *T*-test. The shown data demonstrate the mean ± SEM of the results unless otherwise stated. The statistical significance in all analyses was determined using a confidence interval >95% (*p* < 0.05).

## Results

### Parasitological examination and worm recovery

**Figure 1:**
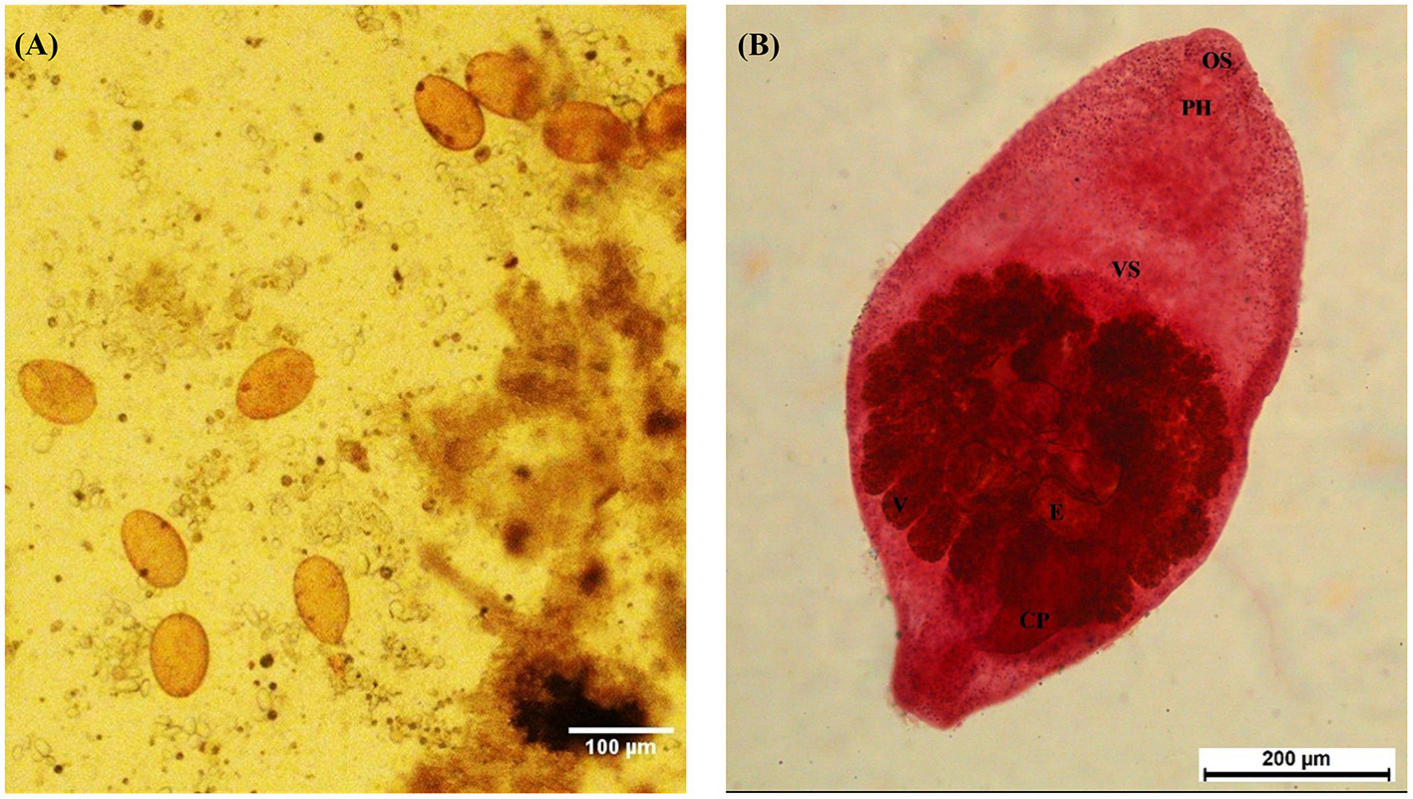
Morphology of *Prohemistomum vivax* eggs and adults recovered from experimentally infected pigeons. **(A)** *P. vivax* eggs isolated from pigeon feces by the sedimentation method. The scale bar was 100 μm. **(B)** Adult *P. vivax* stained with Semichon's acetocarmine stain at 100 × magnification. OS, oral sucker; PH, pharynx; VS, ventral sucker; V, vitellaria; E, egg; CP, cirrus pouch.

In preliminary experiments, high inoculation doses (1,000 and 2,000 metacercariae/pigeon) resulted in severe offensive greenish mucoid diarrhea 4 dpi. Pigeon mortality began at 5 dpi, and by 7 dpi, all birds died. However, 500 metacercariae per host infection did not affect pigeon survival, and pigeons only showed mild diarrhea and gradual weight loss. A 100% of challenged birds were infected. The eggs firstly appeared in feces on the fourth day post-infection. Eggs were large, oval-shaped, yellow, and measured 80–90 μm long by 50–62 μm wide (Figure 1A). On post-mortem examination, we mainly detected flukes in the upper intestine (duodenum and jejunum) of pigeons. Oral infection with 500 metacercariae resulted in the highest worm recovery rate (66.6 ± 5.2%) at 2 dpi, with an average of 333.3 ± 19.1 worms/ pigeon. The recovery rate gradually decreased as the infection progressed, with the lowest recovery rate (14.3 ± 1.7%, 71.7 ± 6.2 worm/ pigeon) observed in the 28-dpi group. Generally, there was a highly significant difference (*p* < 0.0001) in the worm recovery at different time points of infection. Multiple comparisons of the number of worms recovered per bird and recovery rate % at each time point showed a significant change between all groups, except for G2 vs. G3 and G4 vs. G5 comparisons (Table 2).

**Table 2:** Worm recovery from experimentally infected pigeons at different time points.

**Group**	**dpi**	**Worm No./Pigeon**	**Recovery rate (%)**
G1	2	333.3 ± 19.1^a^	66.6 ± 5.2^a^
G2	4	275.7 ± 21.4^b^	55.1 ± 4.7^b^
G3	7	263.7 ± 12.3^b^	52.7 ± 3.4^b^
G4	14	142.7 ± 5.9^c^	28.5 ± 1.6^c^
G5	21	114.3 ± 3.8^c^	22.9 ± 1.1^c^
G6	28	71.7 ± 6.2^d^	14.3 ± 1.7^d^

Data are expressed as the mean ± S.E. of results from at least three birds analyzed per group.

Superscript different letters refer to statistical significance at p < 0.05 in the same column.

### Morphological identification of parasites

Based on morphological features, we identified the recovered flukes as *Prohemistomum vivax* (Sonsino, 1892). The body was undivided into two distinct regions, pyriform or oval, attenuated at both ends and wide at the middle part, concave ventrally. The oral sucker was round and subterminal, leading to a well-developed muscular pharynx. The ventral sucker was well-developed and spherical. The esophagus was short and bifurcated away from the ventral sucker into two intestinal caeca, terminating posterior to the posterior testes. Testes were tandem in position, the anterior one was smooth and ovoid, and the posterior one was quadrilateral. The cirrus pouch was well-developed and on the left side in the caudal region. The ovary was nearly pyramidal, situated laterally between two testes. Vitelline follicles were moderately large, confined in a horseshoe manner around the gonads, and postero-lateral to the tribocytic organ. The caudal appendage and vaginal sphincter were absent. The genital pore was subterminal (Figure 1B). Each organ of *P. vivax* adults was measured and compared to previous reports (Table 3). Statistical analyses of the measurement data showed significant differences in some parameters between this study and previous studies.

**Table 3:** Comparison of *Prohemistomum vivax* trematode morphometric data. Measurements are given in micrometers.

**Parameter**	**Present authors**	**Odhner (30)**	**Dubois (31)**	**Nasr (3)**	**Fahmy and Selim (32)**	**El- Naffar et al (33)**	**Alghabban (34)**	***P*-value**
Body L	622.2–993.9 (833.5 ± 118.7)^b^	750–1,000^b^	810–1,050^b^	740–1,110^b^	1,310–1,550 (1,400) ^a^	1,144–1,910 (1,568)^a^	1,100–1,510^a^	0.003**
Body W	435.4–526.8 (471 ± 37.9)^c^	450–650^bc^	570–680^abc^	520–610^bc^	660–780 (770)^a^	520–820 (717)^ab^	470–690^abc^	0.052
Oral sucker L	34.4–53.1 (42.4 ± 8.1)^c^	70^abc^	60–81^ab^	52–97^a^	77.5–86.8 (82.3)^a^	33–66 (46)^bc^	50–70^abc^	0.04*
Oral sucker W	43.1–64.99 (56.5 ± 9.9)^c^	85^ab^	77–89^ab^	65–77^bc^	89.9–102.3 (95.1)^a^	66–99 (88)^ab^	60–80^bc^	0.008**
Ventral sucker L	29.5–52.5 (43.5 ± 8.2)^a^	NA	NA	NA	NA	44–77 (61)^a^	30–45^a^	0.131
Ventral sucker W	40.9–67.8 (55 ± 11)^a^	NA	NA	NA	NA	44–71 (59)^a^	43–48^a^	0.437
Pharynx L	29.5–52.7 (38.7 ± 8)^b^	60^ab^	60^ab^	58–77^a^	55.8–68.2 (60.6)^ab^	40–70 59^ab^	42–72^ab^	0.29
Pharynx W	41.7–61 (47.7 ± 10)^c^	85^ab^	93^a^	58–77^abc^	55.8–71.3 (64.1)^abc^	55–77 (66)^abc^	40–80^bc^	0.149
Cirrus pouch L	260.2–302.3 (291.5 ± 19.5)^b^	NA	240–315^b^	NA	279.3–372 (336.8)^b^	450–630 (570)^a^	NA	<0.001***
Cirrus pouch W	37.6–72.2 (57.5 ± 11.6)^b^	NA	135^a^	13–26^b^	80.6–126.4 (104.5)^a^	99–155 (122)^a^	NA	0.001**
Cirrus pouch L	2–5 (3.3 ± 1)^abc^	4–5^a^	NA	1–3^c^	2–3^bc^	3–5 4^ab^	1–3^c^	0.019*
Ova L	74.1–91.8 (80.1 ± 10.2)^b^	100^ab^	100–108^a^	90–103^ab^	90–93 (91.5)^ab^	77–99 (82.5)^b^	75–90^c^	<0.001***
Ova W	39.1–69.8 (56.2 ± 11.3)^a^	60^a^	60–65^a^	45–77^a^	65–68.2 (66)^a^	44–66 (47.5)^a^	40–65^a^	0.699

Our measurement data are represented as range (mean ± SD).

L, length; W, width; NA, not available; No., number; Within each raw, values with superscript similar letters had no statistically significant differences; ^*^p < 0.05; ^**^p < 0.01; ^***^p < 0.001.

### Molecular identification and phylogenetic analysis

We successfully amplified ITS1 partial sequence, 5.8S rRNA complete sequence, and ITS2 partial sequence from *P. vivax*. The amplified fragment produced a specific band at about 1,000 bp, consistent with the expected size. Sequence analysis showed that the amplified ITS fragment from *P. vivax* was 1,158 bp, comprising a part of ITS1 (678 bp), 5.8S rRNA (157 bp), and a part of ITS2 (323 bp). The amplified fragment sequence was uploaded into GenBank (Accession No. ON775468).

**Figure 2:**
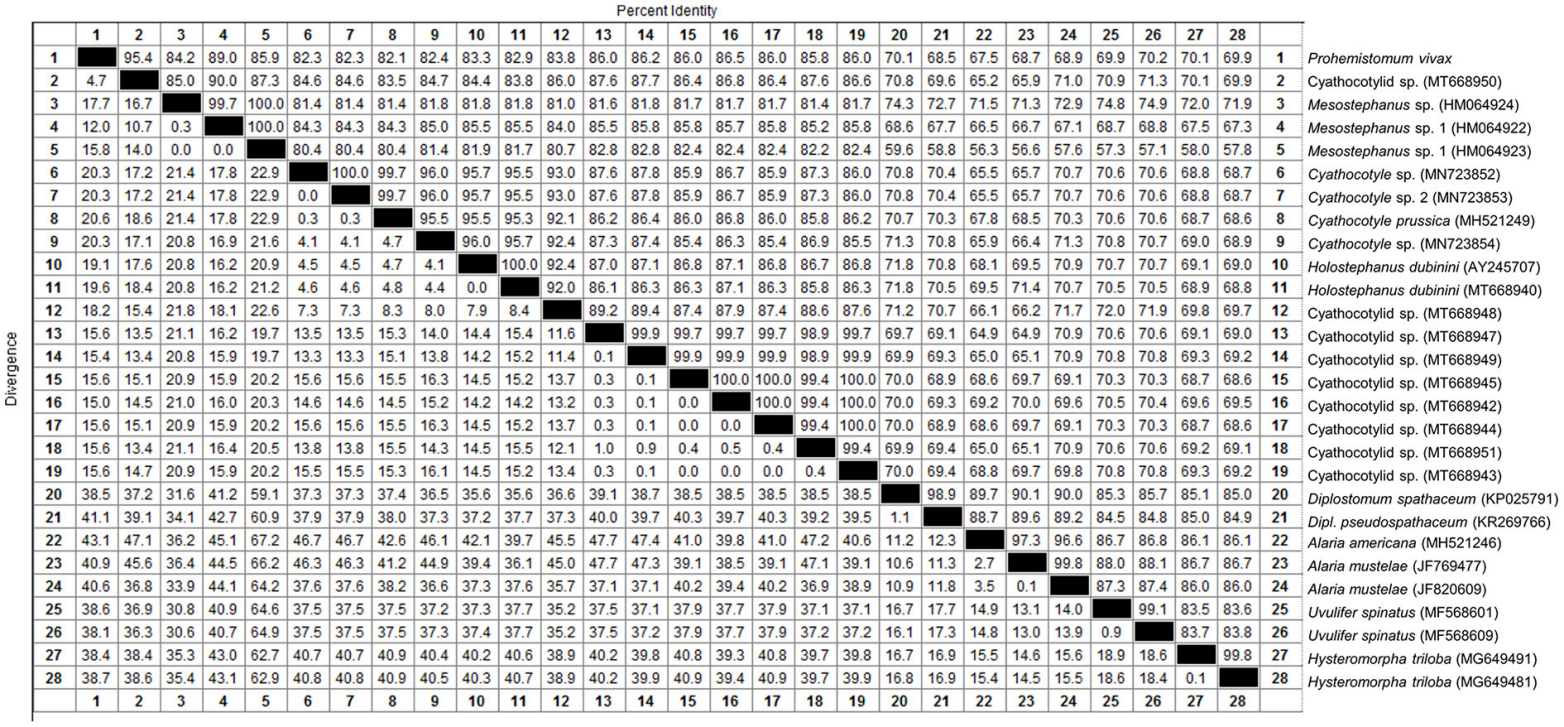
Pairwise distance comparison of IT1, 5.8S, and IT2 region of *Prohemistomum vivax* from experimentally infected pigeons to that of other Diplostomata trematodes.

**Figure 3:**
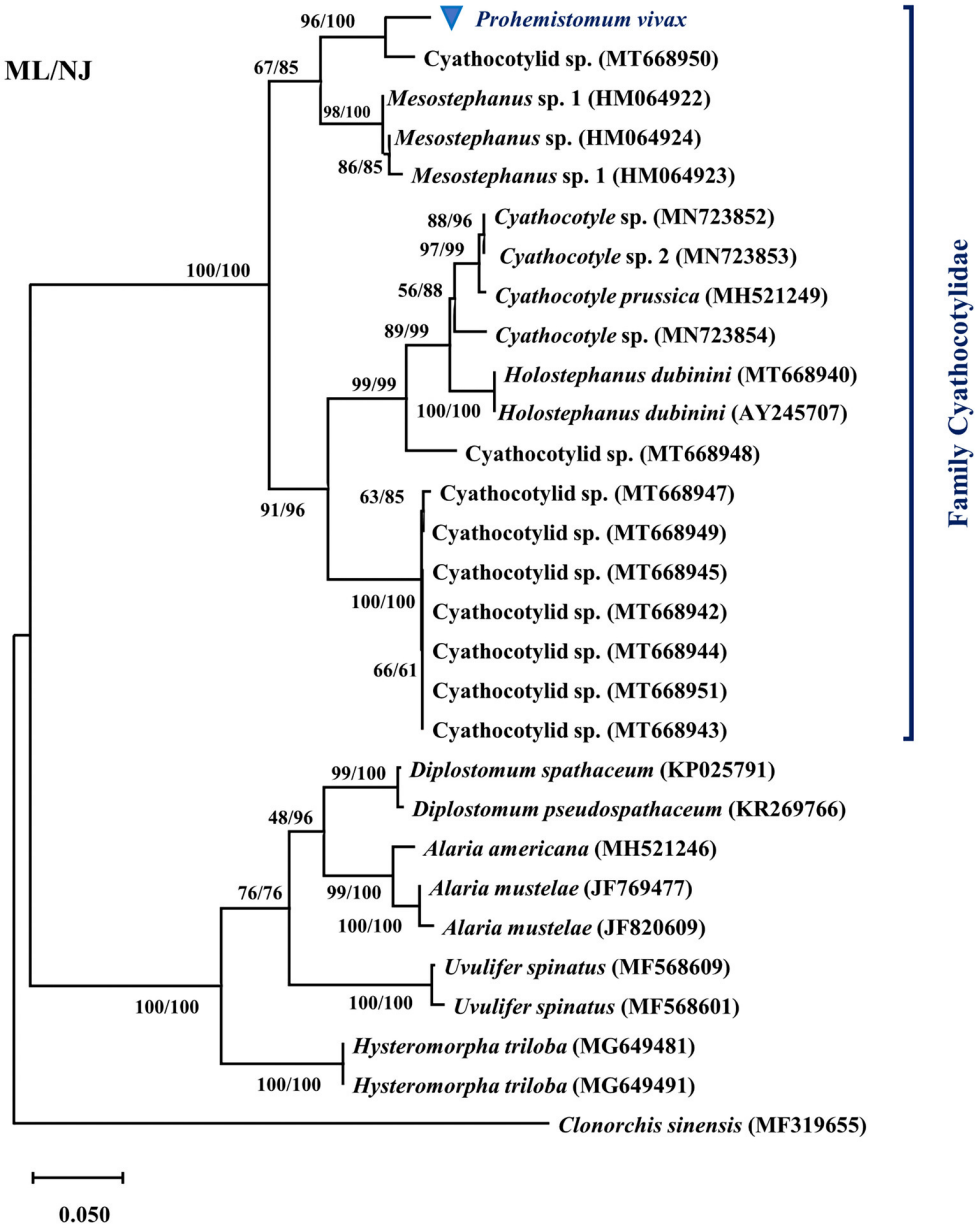
Phylogenetic tree based on the ITS1-5.8S-ITS2 region of *Prohemistomum vivax* and other Diplostomata by the maximal likelihood (ML) and neighbor-joining (NJ) methods.

We determined the pairwise distance of the ITS1-5.8S-ITS2 region of *P. vivax* with those of 27 Diplostomata trematode sequences in GenBank (Figure 2). The Maximum Likelihood and Neighbor-joining phylogenetic analysis (Figure 3) had the same topology. Based on phylogenetic analysis and the pairwise distance comparison of our sample to the related sequences, our sample (*P. vivax*) belonged to a clade supported by 100% bootstrap, representing the trematode family Cyathocotylidae. *Prohemistomum vivax* was most closely related to cyathocotylid metacercariae isolated from common carp (*Cyprinus carpio*) fish from Hungary (MT668950) with an identity percent of 95.4%. Both samples clustered in one clade supported by a high bootstrap value (96/100%, ML/NJ). This clade clustered with that containing *Mesostephanus* sp. metacercariae isolates (HM064922-HM064924) from the pumpkinseed (*Lepomis gibbosus*) fish in Canada, with an identity percent (84.2–89%) to our sample.

### Histopathological evaluation

**Figure 4:**
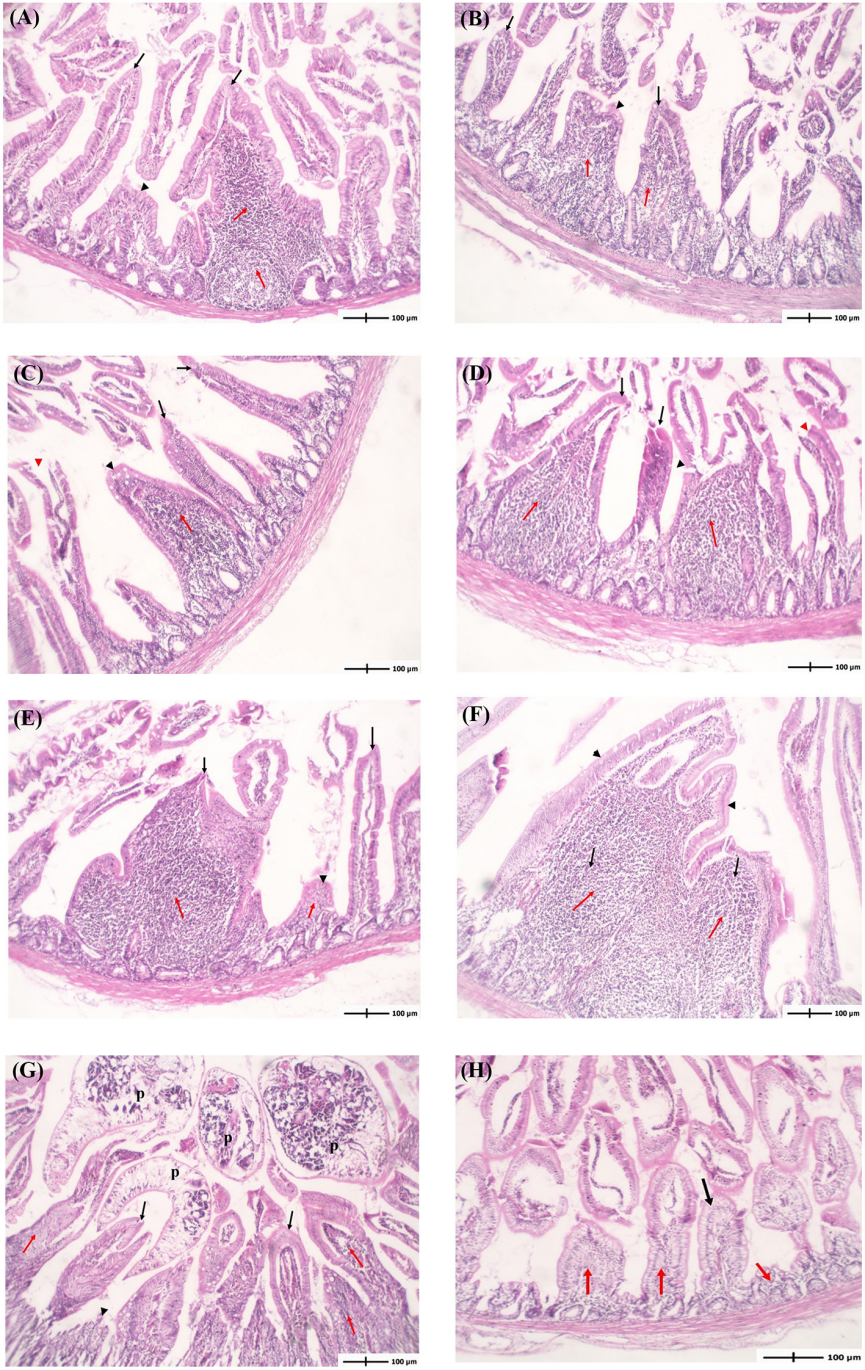
Histopathological examination of the intestine of pigeons infected with *Prohemistomum vivax*. **(A–F)** Histopathological lesions in pigeons from 2-, 4-, 7-, 14-, 21-, and 28-dpi 1-6 groups, respectively. Intestinal tissues from groups 1-6 revealed variable extent of inflammatory cells infiltration in mucosal lamina propria (red arrows). Some villi are uniform (black arrows), others show shortening, thickening, or atrophy (black arrowheads), and others show focal surface ulceration (red arrowheads). **(G)** Adult flukes (p) entrapping intestinal villi. **(H)** Intestinal tissue from the negative control group revealed normal uniform intestinal villi (black arrows) with lamina propria showing few inflammatory cells (red arrowheads) and rounded uniform glands. In all figures, intestinal tissue sections were stained with H&E and at 100 × magnification.

Histopathological evaluation of intestinal sections from infected pigeons at different time points showed that *P. vivax* induced histopathological lesions as early as 2 dpi. The intensity of lesions progressively increased with the infection course (Figure 4, Table 4). Intestinal tissues from groups 1 to 6 revealed a variable degree of villi shortening and thickening or atrophy, and inflammatory cells infiltration in mucosal lamina propria. These alterations gradually accelerated as the infection progressed from 2 to 28 dpi, with the appearance of fused villi at 21 and 28 dpi (Figures 4A–F). Focal epithelial ulcerations were also detected in intestinal sections at time points following fluke maturity, in 7 and 14 dpi groups (Figures 4C,D). Flukes were seen between the intestinal villi and in the villi interspace without invading the crypt region. Some flukes were attached to the intestinal mucosa, pinching intestinal villi (Figure 4G). Intestinal tissues from uninfected pigeons (NC group) revealed normal long and slender uniform intestinal villi. Lamina propria occasionally showed few inflammatory cells with rounded uniform glands (Figure 4H). We used four parameters to evaluate the histopathological changes in pigeon intestines during infection (Table 4). The values of intestinal histological score gradually increased with the infection course, with the highest score (6) detected in 14-, 21-, and 28-dpi groups. The intensity of inflammatory infiltration and villous alterations gradually accelerated as the infection progressed from 2 to 28 dpi, with the induction of villous fusion at 21 and 28 dpi. Moreover, the involvement percentage increased from up to 25% in the 2-dpi group to 51–75% in the 21- and 28-dpi groups (Table 4).

**Table 4:** Histopathological evaluation of intestinal sections from *Prohemistomum vivax*-infected pigeons.

**Group**	**Inflammation with villus atrophy**	**Inflamed area/extent**	**Surface ulceration**	**Involvement percentage**	**Total histological score**
NC	0	0	0	0	0
1	1	1	0	1	3
2	1	1	0	2	4
3	1	1	1	2	5
4	2	1	1	2	6
5	2	1	0	3	6
6	2	1	0	3	6

Group 1–6 were euthanized at 2, 4, 7, 14, 21, and 28 dpi, respectively.

### Cytokine gene expression in intestines

**Figure 5:**
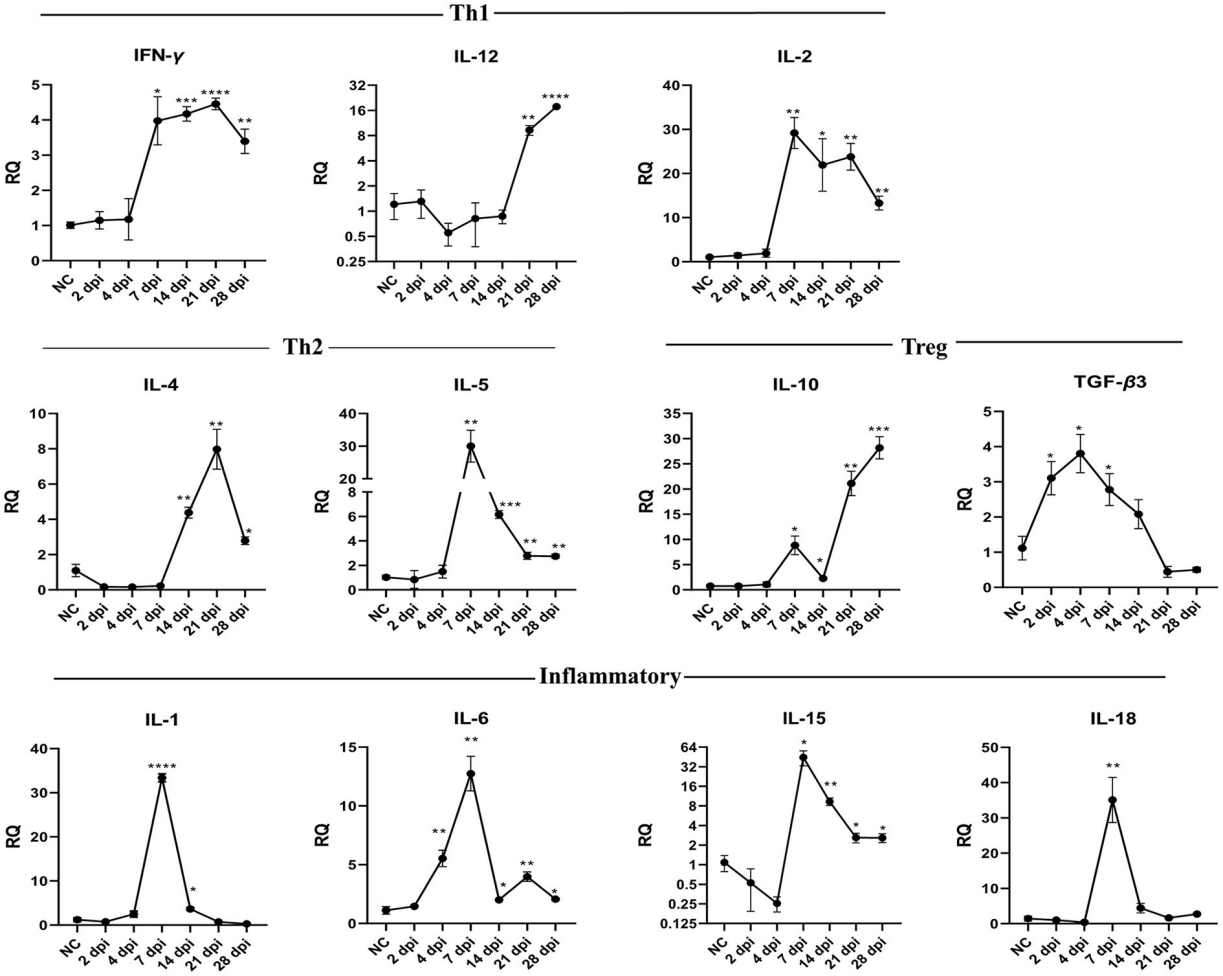
Temporal changes of the cytokine mRNA expressions in the intestine of pigeons experimentally infected with *Prohemistomum* vivax. The X-axis represents days post-infection (dpi), and NC refers to negative control uninfected pigeons. The Y-axis represents relative quantities (RQ) of cytokine expression after normalization with β-actin based on the 2^−Δ*ΔCT*^ method. Error bars show SEMs. Significant differences between each time-point and negative control (NC) uninfected group: *p < 0.05; **p < 0.01; ***p < 0.001, or ****p < 0.0001 (using Student *T*-test).

To understand how the immune response altered throughout the infection, we compared the gene expression of eleven cytokines in the intestinal tissues of infected and uninfected pigeons using RT-qPCR. Results showed that *P. vivax* infection significantly increased Th1, Th2, Treg, and inflammatory cytokines gene expression (Figure 5, Table 5). Cytokines transcriptional trends revealed an immunosuppressive response during early infection stages, with decreased expression of some cytokines, such as IL-12, IL-4, IL-5, IL-1, IL-18, and IL-15, at 2 dpi, 4 dpi, or both compared to control. We did not detect any significant change in Th1 cytokines (IFN-γ, IL-12, and IL-2) gene expression during early infection. Increased IFN-γ mRNA signals were detected in small intestine 7 dpi (*p* = 0.0127) and peaked at 21 dpi (*p* < 0.0001). IL-12 mRNA signals increased in late infection during days 21–28 dpi. IL-2 significant expression levels were detected at 7–28 dpi, peaking at 7 dpi. IL-10 mRNA levels in the intestine showed a significantly higher expression than the control in late infection from 7 to 28 dpi, peaking at 28 dpi. TGF-β3 mRNA in intestine was rapidly elevated at 2 dpi (*p* = 0.0265), peaked at 4 dpi (*p* = 0.0135), remained significantly high until 7 dpi (*p* = 0.0418) followed by a non-significant change between 14 and 28 dpi compared to non-infected group. Fluke infection also upregulated Th2 cytokines (IL-4 and IL-5). IL-4 expression was significantly higher in infected groups than in the control group at 14–28 dpi, with the peak value recorded at 21 dpi (7.9-fold-change, *p* = 0.0044). IL-5 was significantly overexpressed at 7–28 dpi. IL-5 upregulation peaked at 7 dpi and then decreased toward late infection at 28 dpi, from 30 to 2.7 times the control. IL-1 inflammatory cytokine was significantly upregulated at 7 dpi (33-fold-change, *p* < 0.0001) in infected pigeons compared to control, remained high at 14 dpi (3.6-fold-change, *p* = 0.0101), and then decreased to basal level. A significantly higher expression level of IL-18 was only detected at 7 dpi (35-fold-change, *p* = 0.0064). IL-6 transcription level significantly increased in the period from 4 to 28 dpi, with the highest level recorded at 7 dpi (12.8-fold-change, *p* = 0.0015). IL-15 upregulation was observed at 7–28 dpi, with a gradual decrease toward the end of the experiment.

**Table 5:** Relative cytokine gene expression in the intestine of *Prohemistomum vivax-*experimentally infected pigeons compared to the control group.

**Target gene**	**2 dpi**		**4 dpi**		**7 dpi**		**14 dpi**		**21 dpi**		**28 dpi**	
	**RQ**	***P***	**RQ**	***P***	**RQ**	***P***	**RQ**	***P***	**RQ**	***P***	**RQ**	***P***
IFN-γ	1.149	0.6211	1.178	0.7894	3.977	0.0127	4.173	0.0001	4.455	<0.0001	3.394	0.0026
TGF-β3	3.105	0.0265	3.805	0.0135	2.778	0.0418	2.08	0.1436	0.4437	0.1411	0.5022	0.1449
IL-1	0.7504	0.3769	2.477	0.1997	33.38	<0.0001	3.651	0.0101	0.7139	0.3116	0.2754	0.0925
IL-2	1.414	0.5651	1.933	0.3915	29.17	0.0013	21.93	0.0249	23.79	0.0017	13.28	0.0015
IL-4	0.17	0.0589	0.1596	0.0578	0.2332	0.07	4.379	0.0022	7.976	0.0044	2.786	0.0149
IL-5	0.8516	0.8228	1.491	0.4484	30.01	0.0041	6.165	0.0001	2.783	0.0067	2.748	0.0021
IL-6	1.461	0.3622	5.534	0.0045	12.75	0.0015	2.009	0.0497	3.98	0.0051	2.066	0.0478
IL-10	0.7516	>0.9999	1.062	0.6213	8.81	0.0126	2.25	0.0104	21.1	0.0011	28.16	0.0002
IL-12	1.309	0.8848	0.55	0.216	0.8173	0.5541	0.8712	0.4916	9.287	0.0037	17.74	<0.0001
IL-15	0.5265	0.2857	0.2539	0.0555	44.52	0.0196	9.348	0.0031	2.603	0.0466	2.587	0.0399
IL-18	1.016	0.5958	0.406	0.2412	35.1	0.0064	4.418	0.1207	1.661	0.7688	2.745	0.1386

The relative quantities (RQ) were calculated using the 2^−Δ*ΔCT*^ method. P-values represent statistical significance at p < 0.05 compared to controls.

### Cytokine gene expression in spleens

**Figure 6:**
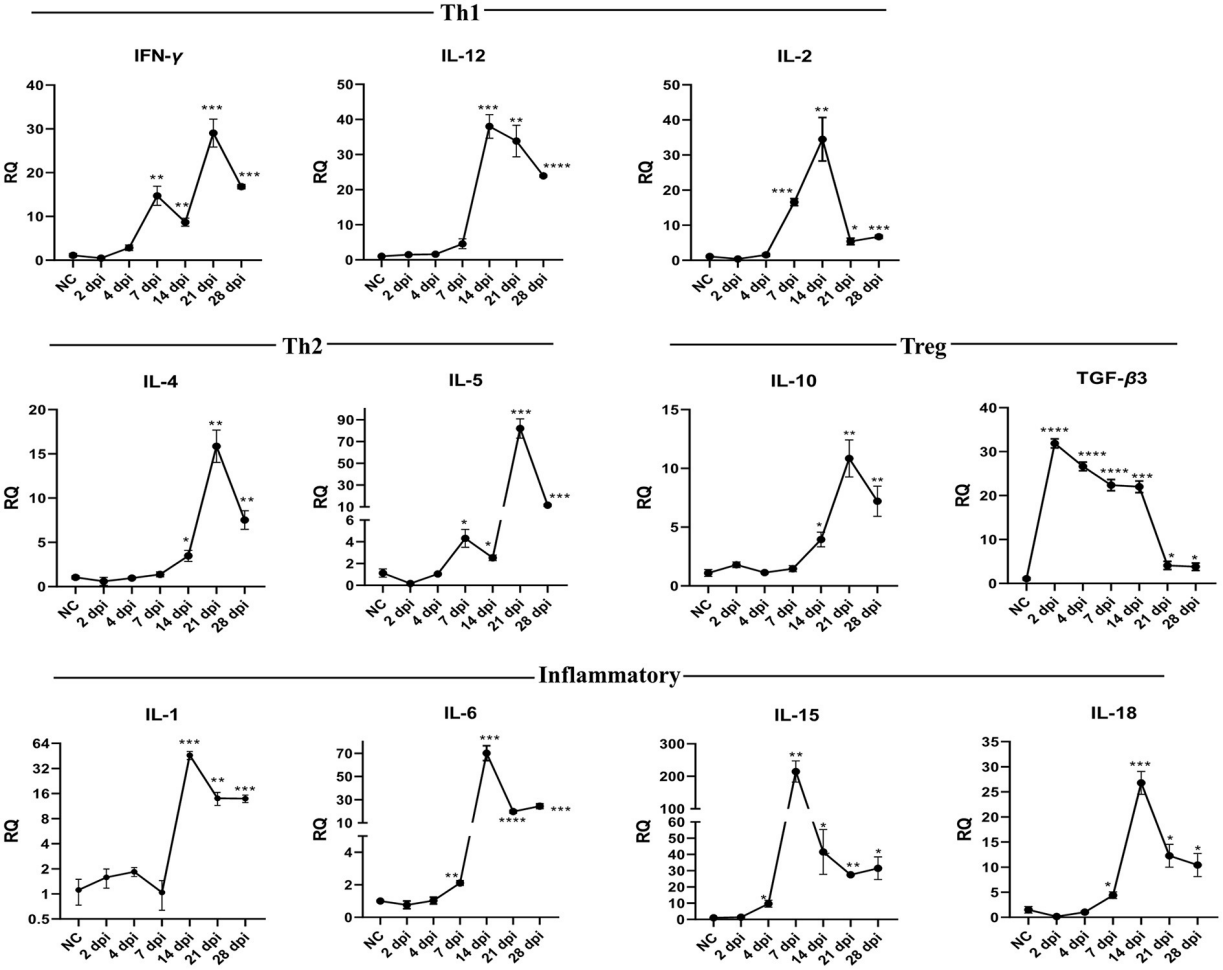
Temporal changes of the cytokine mRNA expressions in the spleen of pigeons experimentally infected with *Prohemistomum* vivax. The X-axis represents days post-infection (dpi), and NC refers to negative control uninfected pigeons. The Y-axis represents relative quantities (RQ) of cytokine expression after normalization with β-actin based on the 2^−Δ*ΔCT*^ method. Error bars show SEMs. Significant differences between each time-point and negative control (NC) uninfected group: *p < 0.05; **p < 0.01; ***p < 0.001, or ****p < 0.0001 (using Student *T*-test).

Figure 6 and Table 6 show changes in cytokine mRNA expressions in the spleen of *P. vivax*-infected pigeons. Increased IFN-γ mRNA signals were detected at 7–28 dpi, peaking at 21 dpi (29-fold-change, *p* = 0.0010). IL-12 mRNA signals significantly increased during days 14–28 PI, with the highest value recorded at 14 dpi and the lowest detected at 28 dpi. IL-2 cytokine expression significantly increased at 7–28 dpi. A significant increase in IL-10 mRNA levels was detected late at 14 dpi, with the peak (10.8 times that of the control group) occurring at 21 dpi (*p* = 0.0036). TGF-β3 mRNA was significantly elevated throughout the infection from 2 to 28 dpi. However, the fold change of upregulation peaked at 2 dpi (31.9-fold-change, *p* < 0.0001) and gradually decreased as the infection progressed. Th2 cytokines expression significantly increased at 7–28 dpi for IL-5 and 14–28 dpi for IL-4. The peak expression level was detected at 21 dpi for IL-4 and IL-5. IL-1 cytokine was significantly upregulated at 14–28. Significantly higher expression levels of IL-6 and IL-18 were found at 7–28 dpi. IL-15 upregulation was observed at 4–28 dpi.

**Table 6:** Relative cytokine gene expression in the spleen of *Prohemistomum vivax-*experimentally infected pigeons compared to the control group.

**Target Gene**	**2 dpi**		**4 dpi**		**7 dpi**		**14 dpi**		**21 dpi**		**28 dpi**	
	**RQ**	***P***	**RQ**	***P***	**RQ**	***P***	**RQ**	***P***	**RQ**	***P***	**RQ**	***P***
IFN-γ	0.484	0.235	2.857	0.0847	14.72	0.0036	8.699	0.0017	29.04	0.001	16.81	<0.0001
TGF-β3	31.89	<0.0001	15.93	<0.0001	22.39	<0.0001	22.01	0.0001	39.01	0.0001	76.14	<0.0001
IL-1	1.58	0.4541	1.845	0.1763	1.04	0.8983	46.06	0.0009	14.01	0.0069	13.91	0.001
IL-2	0.376	0.066	1.567	0.3667	16.59	0.0001	34.5	0.0057	5.399	0.0108	6.71	0.0004
IL-4	0.594	0.4117	0.950	0.727	1.375	0.3847	3.467	0.0201	15.86	0.0013	7.528	0.0036
IL-5	0.164	0.0642	1.047	0.8426	4.323	0.0243	2.528	0.0365	82.03	0.0008	11.53	0.001
IL-6	0.764	0.3775	1.035	0.8806	2.113	0.0014	70.12	0.0004	19.72	<0.0001	24.46	0.0003
IL-10	1.794	0.1338	1.129	0.9174	1.461	0.3968	3.946	0.0145	10.84	0.0036	7.202	0.0096
IL-12	1.471	0.1816	1.611	0.1282	4.577	0.0636	38.03	0.0004	33.85	0.0019	23.92	<0.0001
IL-15	1.41	0.5091	9.681	0.0172	214.9	0.0028	41.58	0.0422	27.49	<0.0001	31.52	0.0121
IL-18	0.149	0.1026	1.043	0.537	4.399	0.0341	26.8	0.0004	12.26	0.0104	10.41	0.02

The relative quantities (RQ) were calculated using the 2^−Δ*ΔCT*^ method. P-values represent statistical significance at p < 0.05 compared to controls.

### Correlation analysis of Th1/Th2/Treg cytokines

Pearson's correlation analysis was conducted on each pair of cytokines based on relative expression quantity (RQ) from infected groups (Table 7). In the intestine, this analysis showed a significant positive association between IFN-γ mRNA expression and IL-2 (*r* = 0.86, *p* < 0.0001) and Th2 cytokine (IL-4: *r* = 0.61, *p* = 0.0072) as well as between IL-2 and IL-5 (*r* = 0.64, *p* = 0.0042). IL-10 gene expression was significantly associated with both Th1 (IFN-γ: *r* = 0.48, *p* = 0.0461; IL-12: *r* = 0.92, *p* < 0.0001) and Th2 cytokines (IL-4: *r* = 0.49, *p* = 0.0380). TGF-β3 relative mRNA expression demonstrated a significant negative correlation to the other cytokines (IFN-γ: *r* = −0.65, *p* = 0.0032; IL-12: *r* = −0.76, *p* = 0.0002; IL-4: *r* = −0.70, *p* = 0.0011; IL-10: *r* = −0.79, *p* < 0.0001).

**Table 7:** Pairwise correlation coefficients between Th1/Th2/Treg cytokines in the intestine (above) and spleen (below) of pigeons experimentally infected with *Prohemistomum vivax*.

	**IL-10**	**IFN-γ**	**IL-12**	**IL-2**	**IL-4**	**IL-5**	**TGF-β3**
IL-10	1	0.475*	0.915**	0.322	0.492*	−0.079	−0.791**
IFN-γ	0.763**	1	0.301	0.864**	0.610**	0.439	−0.654**
IL-12	0.658**	0.613**	1	0.076	0.421	−0.288	−0.762**
IL-2	−0.051	0.043	0.499*	1	0.463	0.643**	−0.403
IL-4	0.909**	0.857**	0.658**	−0.117	1	−0.265	−0.704**
IL-5	0.833**	0.823**	0.508*	−0.191	0.936**	1	0.139
TGF-β3	−0.856**	−0.829**	−0.625**	0.024	−0.841**	−0.676**	1

^*^Correlation is significant at the 0.05 level (2-tailed); ^**^Correlation is significant at the 0.01 level (2-tailed).

In spleens, a strong positive correlation was also detected between IFN-γ and Th2 cytokine expressions (IL-4: *r* = 0.86, *p* < 0.0001; IL-5: *r* = 0.82, *p* < 0.0001). IL-12 mRNA was positively correlated to Th2 cytokines (IL-4: *r* = 0.66, *p* = 0.003; IL-5: *r* = 0.51, *p* = 0.0315) and other Th1 cytokines (IFN-γ: *r* = 0.61, *p* = 0.0068; IL-2: *r* = 0.50, *p* = 0.0352). A strong positive correlation was detected between IL-4 and IL-5 (*r* = 0.94, *p* < 0.0001). IL-10 expression in spleen was positively correlated with both Th1 and Th2 cytokines (IFN-γ: *r* = 0.76, *p* = 0.0002; IL-12: *r* = 0.66, *p* = 0.0030; IL-4: *r* = 0.91, *p* < 0.0001; IL-5: *r* = 0.83, *p* < 0.0001). The relative mRNA expression of TGF-β3 was negatively correlated to other cytokines (IFN-γ: *r* = −0.83, *p* < 0.0001; IL-12: *r* = −0.62, *p* = 0.0056; IL-4: *r* = −0.84, *p* < 0.0001; IL-5: *r* = −0.68, *p* = 0.0021; IL-10: *r* = −0.86, *p* < 0.0001), in Table 7.

### Comparing cytokine expression in the intestine and spleen of *P. vivax*-infected pigeons

Table 8 compares relative cytokine expressions between the intestine and spleen of *P. vivax*-infected pigeons. Our findings revealed a significant difference in the relative quantity of cytokines between the two organs throughout the infection. Th1 (IFN-γ and IL-12), IL-4, TGF-β3, and IL-15 cytokines had significantly higher expression in the spleen than in the intestine. However, at 7, 21, and 28 dpi, IL-2 and IL-10 levels in the intestine were significantly higher than those in the spleen. Other cytokines (IL-5, IL-1, IL-6, and IL-18) were significantly higher in the intestine at early infection but became higher in the spleen as the infection progressed. IL-5 levels were higher in the intestine than in the spleen at 2–14 dpi, with a significant difference at 7–14 dpi. Then, at 21–28 dpi, IL-5 levels in the spleen were higher than in the intestine. The intestine had significantly higher expression levels of IL-1 at 7 dpi, IL-6 at 4–7 dpi, and IL-18 at 2 and 7 dpi, while the spleen had higher levels of all three cytokines at 14–28 dpi.

**Table 8:** Comparison of relative cytokine gene expression in the intestine and spleen of *Prohemistomum vivax-*experimentally infected pigeons.

**Target gene**	**Organ**	**2 dpi**		**4 dpi**		**7 dpi**		**14 dpi**		**21 dpi**		**28 dpi**	
		**RQ**	***P***	**RQ**	***P***	**RQ**	***P***	**RQ**	***P***	**RQ**	***P***	**RQ**	***P***
IFN-γ	Intestine	1.149	0.105	1.178	0.124	3.977	0.009**	4.173	0.009**	4.455	0.002**	3.394	<0.001**
	Spleen	0.484		2.857		14.72		8.699		29.04		16.81	
TGF-β3	Intestine	3.105	<0.001**	3.805	<0.001**	2.778	<0.001**	2.08	<0.001**	0.4437	0.017**	0.5022	0.018**
	Spleen	31.89		15.93		22.39		22.01		39.01		76.14	
IL-1	Intestine	0.7504	0.142	2.477	0.441	33.38	<0.001**	3.651	0.001**	0.7139	0.006**	0.2754	0.001**
	Spleen	1.58		1.845		1.04		46.06		14.01		13.91	
IL-2	Intestine	1.414	0.138	1.933	0.728	29.17	0.026**	21.93	0.218	23.79	0.004**	13.28	0.016**
	Spleen	0.3755		1.567		16.59		34.5		5.399		6.71	
IL-4	Intestine	0.1702	0.398	0.1596	0.005**	0.2332	0.017**	4.379	0.259	7.976	0.022**	2.78693	0.011**
	Spleen	0.5937		0.9503		1.375		3.467		15.86		7.528	
IL-5	Intestine	0.8516	0.402	1.491	0.448	30.01	0.007**	6.165	0.001**	2.783	0.001**	2.748	0.002**
	Spleen	0.1634		1.047		4.323		2.528		82.03		11.53	
IL-6	Intestine	1.461	0.054	5.534	0.004**	12.75	0.002**	2.009	<0.001**	3.98	0.001**	2.066	<0.001**
	Spleen	0.7635		1.035		2.113		70.12		19.72		24.46	
IL-10	Intestine	0.7516	0.056	1.062	0.898	8.81	0.017**	2.25	0.056	21.1	0.023**	28.16	0.001**
	Spleen	1.794		1.129		1.461		3.946		10.84		7.202	
IL-12	Intestine	1.309	0.775	0.55	0.0024**	0.8173	0.061	0.8712	<0.001**	9.287	0.006**	17.74	0.001**
	Spleen	1.471		1.611		4.577		38.03		33.85		23.92	
IL-15	Intestine	0.5265	0.220	0.2539	0.013**	44.52	0.008**	9.348	0.080	2.603	0.001**	2.587	0.015**
	Spleen	1.41		9.681		214.9		41.58		27.49		31.52	
IL-18	Intestine	1.016	0.028**	0.406	0.157	35.1	0.009**	4.418	0.001**	1.661	0.010**	2.745	0.029**
	Spleen	0.1486		1.043		4.399		26.80		12.26		10.41	

^**^Significant difference at *p* < 0.05 using independent samples T-test.

## Discussion

In this study, the morbidity and fatality in infected pigeons depended on the infection dose. The ingestion of 1,000 or more metacercariae resulted in the death of all birds within a week of infection, while 500 metacercariae inoculum did not cause fatalities in pigeons during the studied period of 28 days. Similarly, the severity of the clinical signs and the mortality in *Neodiplostomum seoulense*-experimentally infected mice was proportional to the metacercariae inoculum. Mice receiving 1,000 *N. seoulense* metacercariae died within 16 days post-infection. The possible causes of death might be excessive fluid loss and malnutrition due to malabsorption, mucosal bleeding, and persistent diarrhea (2). The earlier death in our study compared to that recorded with *N. seoulense* might be related to the difference in host and parasite species. We showed that metacercariae could successfully develop into adult *P. vivax* in the intestine of pigeons. This result agreed with previous studies (5, 7). *P. vivax* characteristic eggs were firstly detected in pigeon feces 4 days post-infection, in agreement with previous results in mice and rats (17, 34). Mahfouz et al. (17) suggested that the parasite's early growth may necessitate a rich supply of nutrients. Thus, it is possible to detect the parasite engulfing the epithelial villi. The upper intestine was the primary habitat of *P. vivax* in the final host (pigeons), consistent with results reported in *P. vivax*-infected mice (17, 18).

The recovered flukes were confirmed as *P. vivax* based on combined morphological and molecular identification. Although, there was a significant difference between our sample measurements and that of previous studies in some parameters, our sample matched all morphological characters of *P. vivax* reported in previous studies (3, 30, 32–34). Our sample was smaller than that recorded by Fahmy and Selim (32) and El-Nafffar et al. (33). This difference might be related to the different biological environments in the intestine of variable hosts (animals and birds) (42) and the difference between experimental and natural infection. For example, we compared measurements of *P. vivax* recovered from human (3), naturally infected dogs (32), experimentally infected dogs and cats (33), and experimentally infected rats (34) Similarly, Fahmy et al. (42) showed that *P. vivax* recovered from dogs were larger than that collected from buff-backed herons. We successfully amplified the ITS1, 5.8 S rRNA, and ITS2 region from *P. vivax* for the first time. In the phylogenetic tree, our sample (*P. vivax*) belonged to the family Cyathocotylidae clade. *P. vivax* was closely related to cyathocotylid metacercariae recovered from Hungarian common carp fish (95.4% homology) and clustered in one clade with *Mesostephanus* sp. metacercariae, which might represent the subfamily prohemistominae clade. These findings agreed with our sample morphological description and known taxonomy.

In histopathological examination, we found *P. vivax* flukes between the intestinal villi and never invaded the crypt region, similar to findings in mice (17). *P. vivax* caused histopathological alterations as early as 2 dpi. Furthermore, lesion intensity and intestinal histological score gradually increased with the infection course, with the highest score (6) detected on 14, 21, and 28 dpi. Intestinal tissue lesions included shortening and thickening or atrophy of villi, villous fusion, inflammatory cell infiltration in mucosal lamina propria, and focal epithelial ulcerations. Similar pathological changes were seen in the upper intestine of *P. vivax*-infected pigeons and mice (7, 17), *Heterophyes heterophyes*-infected mice (43), *Echinostoma malayanum*-infected hamsters, rats, mice, and gerbils (44), *Metagonimus yokogawai*-infected cats, *N. seoulense*-infected rats and mice, *Pygidiopsis summa*-infected rats and mice, and *Gymnophalloides seoi*-infected mice, reviewed by Chai (2). The observed pathological alterations might be attributed to the parasite's direct effect and the host's response. Intestinal flukes, including *P. vivax*, can induce mechanical and chemical damage in the host gut. *P. vivax* possesses a spiny tegument and well-developed adhesive tribocytic organ (45), which may directly affect parasite pathogenesis due to irritation of the host mucosa (17). Additionally, the tribocytic organ of diplostomidean flukes can pierce the host villi and secrete alkaline phosphatase that can lyse the villi (2). Early studies proposed that frequent movement and rotation of the trematode anterior part injury the intestinal mucosa and result in mechanical pressure on adjacent villi (46). Chai (47) suggested that the villi pressure atrophy in *M. yokogawai*-infected rat intestine might be related to the accumulation of mucus and gases in the gut lumen.

Previous reports have supported the induction of host protection mechanisms against *P. vivax*, activating the humoral and cellular host immune responses (17). Helal et al. (48) observed a massive influx of eosinophils and mast cells in the intestines of infected rats. Still, no studies have investigated the molecular pathology and the immune effector mechanism of intestinal infection with *P. vivax*. The immune response is regulated by CD4 T cells that are primarily classified into Th1 or Th2 T cells based on the released cytokines. Th1 cells mainly release IFN-γ and IL-12, while Th2 cells release IL-4, IL-5, IL-9, IL-10, and IL-13 (49, 50). These cell subsets induce different and counterregulatory immune responses. Th2 response cytokines regulate the host protection, whereas Th1 responses are related to chronic infections (25). Nevertheless, the preferential stimulation of Th1 and Th2 cells during parasite infections depends on many variables, including host genetic nature, parasite species, infection route and stage, and acute or chronic infection (25, 26).

This study demonstrated that *P. vivax* infection significantly impacted Th1, Th2, Treg, and inflammatory cytokines gene expression. The cytokine transcriptional profile indicated an immunosuppressive response in the early stages, a mixed Th1/Th2 response as the infection progressed, and a late tilting toward Th1/Treg response. At early infection (2–4 dpi), the expression of some cytokines was generally lower than in controls, implying an immunosuppressive condition to facilitate parasite colonization. This early immunosuppression state and cytokine decline were also observed during *Fasciola gigantica* infection and were attributed to the overexpression of the immunosuppressive cytokine TGF-β at 3 dpi (50). In agreement with this hypothesis, our study showed that TGF-β3 mRNA was rapidly elevated in the intestine at 2 dpi and remained significantly high until 7 dpi, followed by a non-significant change at 14–28 dpi. Although TGF-β3 mRNA in the spleen was significantly high throughout the infection period, the fold change of expression peaked at 2 dpi and gradually diminished as the infection progressed. These results suggest that TGF-β3 might play a role in immunosuppression and parasite colonization during early *P. vivax* infection. Similarly, semi-quantitative RT-PCR revealed an upregulation in TGF-β expression in *P. vivax*-infected mice intestines during the examined first 6 days of infection (17). TGF-β was also overexpressed during various parasitic infections, such as *Schistosoma japonicum* (51), *Echinococcus granulosus* (52), and *Heligmosomoides polygyrus* (53). We detected a significant negative correlation between TGF-β3 and other cytokines in both intestines and spleen of infected groups. Although there was no significant difference, TGF-β was also inversely related to other cytokines during *F. gigantica* infection (50). This negative correlation might be related to the immunosuppressive role of TGF-β. TGF-β can suppress T-cell proliferation, cytokine production and cytotoxicity, IFN-γ production, MHC class II expression, and reactive oxygen intermediates and nitric oxide release from activated macrophages (54).

Here, as *P. vivax* infection progressed 7–28 dpi, a mixed Th1 and Th2 responses developed in agreement with results of Shin et al. (26), who showed that mixed Th1 and Th2 responses shared in the activation of humoral and cellular immune reactions throughout *N. seoulense* infection in mice. *P. vivax* infection upregulated Th1 (IFN-γ, IL-12, and IL-2) and Th2 cytokines (IL-4 and IL-5). IFN-γ is released from Th1 cells and is linked to proinflammatory cytokines production, inhibiting Th2 responses (26). IL-12 is critical for enhancing cell-mediated immune responses. IL-12 and IL-12R-deficient humans and mice had impaired immune responses and were vulnerable to intracellular pathogen infections. IL-12 plays an important role in Th cell differentiation into the Th1 subset. IL-12 also stimulates IFN-γ release, in synergy with IL-2 or IL-18, and T and NK cell proliferation and cytolytic activity (55). IL-2 was the first cytokine to be molecularly cloned and was identified as a T cell growth factor vital for T cell proliferation and effector and memory cell generation. Subsequent research showed that IL-2 is necessary for maintaining Treg cells, and its absence results in an intense Treg cell deficiency and autoimmunity because IL-2 encourages Treg cell generation, survival, and activity. Therefore, IL-2 serves two contradictory roles: enhancing conventional T cells to boost immune responses and maintaining Treg cells to control immune responses (56). We found that IFN-γ was upregulated in both the intestine and the spleen (at 7 dpi) before IL-12, which may be contradictory. Overexpression of IFN-γ also proceeded IL-12 upregulation in the spleen and mesenteric lymph node of *N. seoulense*-infected mice, but not in the small intestine (26). Although IL-12 is a major inducer of IFN-γ, IL-2 also significantly impacts IFN- expression. These cytokines (IL-12 and IL-2) can induce IFN-γ expression independently. However, they act synergistically to induce large amounts of IFN-γ, particularly from NK cells (57). At 7 dpi, we found IL-2 upregulation in the intestine and spleen, with 29- and 17-fold increases compared to controls, respectively. The overexpressed IL-2 might have induced IFN-γ production either independently or in synergy with the low amount of IL-12 expressed during early infection. IFN-γ and IL-12 expression is coordinated, meaning that IL-12 induces IFN-γ, and IFN-γ induces IL-12 (58). Therefore, when the expression of IFN-γ increased, IL-12 was upregulated, which in turn exaggerated IFN-γ release. IL-4 is produced by Th2 cells and is essential for Th2 cell differentiation, as well as B cell activation, enabling IgE production. This cytokine plays a crucial role in parasite infections, promoting the host's resistance to parasite invasion (59). IL-5 is a cytokine that is involved in the Th2-type inflammatory response in the host and triggers eosinophil production and stimulation (60). In this study, Th1 and Th2 cytokine expressions were positively correlated, consistent with the findings of Shi et al. (50). This positive correlation might be related to the antagonistic functions of both responses and could be essential for immune homeostasis.

Intestinal nematodes, such as *Heligmosomoides polygyrus, Trichuris muris*, and *Nippostrongylus brasiliensis*, can induce strong Th2 responses (20, 24). On the contrary, intestinal trematodes, such as *N. seoulense*, can activate mixed Th1 and Th2 responses, but Th1 cytokine production (IFN-γ and IL-12) was the more potent among them (26). This result agreed with observations for *Echinostoma caproni* (25), which mainly triggered Th1 responses (elevated IFN-γ and IL-12) while also increasing Th2 cytokines (IL-5 and to a lower extent, IL-4). A single injection of anti-IFN-γ antibodies to *E*. *caproni-*infected mice significantly reduced worm burden, suggesting the IFN-γ role in establishing chronic echinostomiasis (25). Host fatalities were recorded in *N. seoulense* and *P. vivax* but not *E*. *caproni* infections. Thus, chronic infection is linked to a Th1 response (25), while mixed Th1 and Th2 responses may contribute to immunopathogenesis that results in host injury and worm expulsion (26). In *N. seoulense-*infected mice spleens and intestines, numbers of macrophages, activated either by Th1 or Th2 cells, were increased at 14–28 dpi (26), the expected period for worm expulsion (46). *In vitro*, rat macrophages eliminated *N. seoulense* through antibody-dependent cellular cytotoxicity. As a result, Shin et al. (26) concluded that combined Th1 and Th2 responses could result in extensive macrophage infiltration in the infected rat spleen and small intestine, enhancing worm ejection and host damage. Although this conclusion might be functional during the infection with *P. vivax*, a diplostomidean fluke related to *N. seoulense*, more studies are required to assess the role of macrophages in *P. vivax*.

Treg cytokine IL-10 mRNA levels were significantly upregulated at 7–28 dpi in the intestine and 14–28 dpi in the spleen. This result agreed with IL-10 upregulation in other helminthic infections, such as *N. seoulense* (26), *E. caproni* (27), *Fasciola gigantica* (50), *Toxocara canis* (61), *Ostertagia ostertagi* (62), *Strongyloides venezuelensis* (63), and *Trichinella spiralis* (64). IL-10 is an anti-inflammatory cytokine released from Treg cells that can reduce the immunopathological damage in the host by suppressing parasite-induced inflammation (65). IL-10 is also critical for Th2 response and induces T cell response shifting to the Th2 type while acting as a suppressive regulator of IL-12 (Th1) and inhibiting Th1 cell differentiation (65, 66). We suggest that IL-10 expression increased during *P. vivax* infection to counteract the high levels of inflammatory cytokines and maintain the Th1/Th2 balance and immune homeostasis, thereby protecting the host from excessive inflammation and tissue pathology. In our study, there was a positive correlation between IL-10 and Th1 and Th2 cytokines, consistent with the results in *F. gigantica*-buffaloes recorded by Shi et al. (50). Increasing IL-10 levels with the concurrent upregulation of Th1 and Th2 cytokines suggest that IL-10 is required for the Th2 response to *P. vivax* infection. Moreover, IL-10 overexpression follows Th1 response rise to hinder the overexaggerated Th1 cell activity, maintaining Th1/Th2 balance and immune homeostasis.

IL-1 inflammatory cytokine was significantly upregulated at 7–14 dpi in the intestine and 14–28 dpi in the spleen. Similarly, the cytokine IL-1β mRNA was overexpressed in the livers of *F. gigantica-*infected buffaloes (50). We also detected a significantly higher IL-18 expression level at 7 dpi in the intestine and 7–28 dpi in the spleen, in agreement with Alhallaf et al. (67), who showed elevated IL-18 secretion in humans and mice infected with *Trichuris* nematodes. The IL-1 family of cytokines (IL-1α, IL-1β, and IL-18) is released from innate immune cells, such as macrophages, monocytes, dendritic cells, endothelial, and epithelial cells, and are essential for initiating and enhancing innate and adaptive immune responses, resisting microbial infections, regulating immune response (67, 68). IL-18 can promote Th2-type immune responses *via* stimulating IL-4 and IL-13 production from basophils in the presence of a second stimulus (IL-3). Additionally, IL-18 plays a vital role in priming NK cells for IFN-γ production and increases its secretion in response to IL-12 (68). IL-1α and IL-1β have many immunomodulatory roles, including activating monocytes and macrophages, stimulating B cell proliferation and differentiation in collaboration with IL-4 and IL-6, and potentiating antibody production (69). Some studies suggest that IL-1α and IL-1β have a physiological impact on Th cell response development. IL-1 can enhance T cell proliferation by stimulating the transcription of IL-2 receptor (70). Moreover, lymph node cells from IL-1α and β-deficient mice failed to differentiate into Th2 cells in the presence of IL-4. Thus, IL-1α and IL-1β are required, along with IL-4, for Th2 response development. *In vivo*, IL-1α and IL-1β were involved in establishing Th2-mediated resistance (expulsion) to nematodes, where IL-1α and IL-1β-deficient mice were vulnerable to chronic *Trichuris muris* infection, with impaired Th2 responses (69). IL-1 has also been essential for IL-12-induced Th1 cell growth (71). Concurrent overexpression of IL-1 and IL-18 with Th1 and Th2 cytokines in *P. vivax* infection might support previous suggestions for their role in developing Th1 and Th2 responses.

We demonstrated that IL-6 transcription levels significantly increased in the intestine at 4-28 dpi and in the spleen at 7-28 dpi. Similar to our results, *E. caproni*-infected rats and mice showed a significant increase in IL-6 in the spleen and intestine (27). IL-6 activates T helper cells, regulates T cell resistance to apoptosis, and modulates the Treg/Th17 cells balance (72). IL-6 acts as a myokine, i.e., a cytokine released from muscle during contraction (27, 73). IL-6 expression was more marked in the intestine of *E. caproni*-infected rats (resistant host) compared to mice (compatible host), suggesting the possible role of IL-6 in intestinal motility in response to intestinal trematode (27). IL-6 upregulation might alter intestinal smooth muscle motility, hindering worm attachment and feeding in the intestine, thus helping worm expulsion during *P. vivax* infection. Additionally, the high expression of Th1 cytokines (IL-1, IL-18, and IL-6), indicating acute inflammatory response, might be linked to the severe inflammatory cell infiltrations observed in the pigeon intestine.

We observed IL-15 upregulation at 7–28 dpi in *P. vivax*-infected pigeons' intestines and 4–28 dpi in spleens. IL-15 is a proinflammatory cytokine involved in the development, proliferation, and activation of several lymphocyte lineages (74). This cytokine acts as a pleiotropic lymphokine that is not expressed in T cells but is derived from many other cells and tissues, including skeletal tissue, muscle, placenta, lung, kidney, heart, epithelial cells, fibroblasts, and monocytes (75). IL-15 belongs to the common cytokine receptor common gamma chain (γ*c)* family of cytokines (with IL-2, IL-4, IL-7, IL-9, and IL-21), playing essential roles in innate and adaptive immunity. IL-15 receptor (IL-15R) shares the β chain and γ chain of IL-2R. Therefore, IL-15 shares some immunological activities with IL-2, such as stimulating CD4+ and CD8+ T-cell proliferation and activation, T helper cells differentiation, B cells antibodies synthesis, natural killer (NK) cell proliferation and maintenance, and CD8+T cells and NK cells cytolytic activity (76). However, IL-15 activities on T cell-mediated immune responses are distinct from those of IL-2 because IL-15 uses a different α-chain from IL-2R and expresses a specific receptor (IL-15RX). IL-15 can suppress the IL-2-mediated T cell responses and Fas-mediated activation-induced cell death (AICD) process. In contrast to IL-2, IL-15 has little effect on Treg activity, but it can compromise Treg function by acting on CD4+T and CD8+T cells (75). IL-15 can boost both Th1 responses by enhancing IFN-γ and TNF-α production and Th2 responses by enhancing IL-4 and IL-5 release (75, 77). Studies have revealed that IL-15 might play a role in resisting intracellular protozoan infections, including *Leishmania braziliensis, Cryptosporidium, Plasmodium*, and *Toxoplasma gondii*, by influencing Th1 or Th2 response development (77–81). Although little is known about the role of IL-15 in helminth infections, some studies indicated that IL-15 plays a role in the immune response against helminth parasites similar to that played in protozoan parasite infections. The mRNA levels of IL-15 in the immunized group slightly exceeded those found in the *O. ostertagi*-infected group (82). Filarial nematode (*Setaria equina*) excretory-secretory antigens stimulated IL-15 production in mice (83).

The immune response was quite different between the intestine and spleen tissues of *P. vivax*-infected pigeons. The Th1 response (IFN-γ and IL-12) in the intestines appeared more persistent until the end of the experiment, while Th2 cytokine (IL-5) expression fold change peaked at 7 dpi and then decreased toward late infection at 28 dpi. However, the general trend of both Th1 and Th2 cytokines in the spleen was nearly similar, significantly overexpressed at 7–28 or 14–28 dpi and peaking at 14 or 21 dpi. Similarly, Trelis et al. (27) recorded a marked Th1 response (IFN-γ) in the intestine and a mixed Th1/Th2 phenotype in the spleen of *E. caproni*-infected high compatible host (mice).

On comparing the relative cytokine quantity in the intestine and spleen, we observed a significant difference in the cytokine expression levels between the intestine and the spleen throughout the infection. Intestinal epithelial cells represent the first barrier of enteric immunity. Helminths cause intestinal tissue damage during feeding, stimulating the release of cytokine alarmins, such as IL-25, IL-33, and thymic stromal lymphopoietin. These alarmins triggers innate lymphoid 2 cells, a major source of the Th2 cytokines (IL-5 and IL-13). As a result, IL-5 and IL-13 levels can rise within hours of helminth infection (84). These findings could explain the early higher expression of IL-5 in the intestine than in the spleen. The lamina propria provides an influential immune cell population, quickly activated after intestinal helminth infection (84). Inflammatory cell infiltration in the lamina propria was detected in the intestine of *P. vivax*-infected pigeons as early as 2 dpi, which could be related to the higher expression of inflammatory cytokines (IL-1, IL-6, and IL-18) in the intestine during early infection. Additionally, helminth excretory/secretory products and activated dendritic cells circulate to several organs, including the spleen, where they contribute to systemic response development. The spleen is a major source of serum immunoglobulins and Th2 cells. Two weeks after infection with *H. polygyrus*, splenomegaly was observed, with augmented type 2 cytokine expression. Thus, the intestinal mucosal response develops first and is accompanied by higher Th2 cytokine levels, whereas the systemic response (spleen) quickly follows the initial enteric response (84). This hypothesis could explain IL-5, IL-1, IL-6, and IL-18 increased expression in the spleen after 2 weeks of infection following the upregulation in the intestine. Although the spleen expressed more IFN-γ, IL-12, IL-4, TGF-3β, and IL-15 than the intestine, the difference was mainly significant after 7 dpi, possibly after the systemic response initiation. Even though not statistically analyzed, the spleen of *N. seoulense*-infected mice had a higher relative quantity of IFN-γ and IL-4 than the intestine (26), similar to our results. The type 2 immune response at the site of intestinal helminth infection maintains the stimulates of Th2 effector cells and T regulatory cells, which may lead to higher IL-10 levels in the intestine than in the spleen. This upregulation was maintained in the intestine till 28 dpi, perhaps to hinder the increased inflammatory cytokine expressions in the intestine and protect the host from pathological damage. Like IL-10, IL-2 was highly expressed in the intestine compared to the spleen, which might be related to IL-2 role in maintaining Treg cell survival and activity.

In summary, this study is the first to molecularly identify *P. vivax* and illustrate the correlation between the intestinal pathology and the expression of Th1, Th2, Treg, and inflammatory cytokines during experimental *P. vivax* infection. Cytokine expression profiles displayed an early immune suppression state between 2 and 4 dpi, with overexpressed TGF-β3 levels, to enable parasite colonization. A mixed Th1/Th2 immune response developed as the infection progressed, which may be related to host pathological damage and worm expulsion. Treg IL-10 was upregulated during late infection, perhaps to combat the coincided inflammatory cytokines (IL-1, IL-18, and IL-6) high levels and intense pathological lesions, protect the host from excessive tissue damage, and contribute to the Th1/Th2 balance and immune homeostasis.

## Data availability statement

The datasets presented in this study can be found in online repositories. The names of the repository/repositories and accession number(s) can be found in the article.

## Ethics statement

The animal study was reviewed and approved by the Animal Care and Use Committee of the Faculty of Veterinary Medicine, Suez Canal University (Approval No. 2022002).

## Author contributions

AA and GL contributed to the conception and design of the study. AA, MH, YH, LH, and TZ contributed to experiments. AA and MH performed the statistical analysis. AA wrote the first draft of the manuscript. GL supervised the study, analyzed data, and revised the manuscript. All authors read and approved the submitted version.

## Funding

AA was supported by a full Ph.D. scholarship (CSC No. 2018DFH011545) from the Chinese government, and this study was funded by the National Natural Science Foundation of China (Grant No. 31672541).

## Conflict of interest

The authors declare that the research was conducted in the absence of any commercial or financial relationships that could be construed as a potential conflict of interest.

## Publisher's note

All claims expressed in this article are solely those of the authors and do not necessarily represent those of their affiliated organizations, or those of the publisher, the editors and the reviewers. Any product that may be evaluated in this article, or claim that may be made by its manufacturer, is not guaranteed or endorsed by the publisher.

## References

[1] Niewiadomska K. Family Cyathocotylidae Mühling, 1898. In: Gibson D, Jones A, Bray R, editors. Keys to the Trematoda 1. London: CABI: International and the Natural History Museum (2002). p. 201–14. 10.1079/9780851995472.0201

[2] Chai J-Y. Miscellaneous zoonotic species. In: Human Intestinal Flukes. Dordrecht: Springer (2019). p. 491–520. 10.1007/978-94-024-1704-3_9

[3] Nasr M. The occurrence of *Prohemistomum vivax* (Sonsino, 1892) Azim, 1933. Infection in man, with a redescription of the parasite. Lab Med Prog. (1941) 2:135–149.

[4] Abou-Eisha A Saleh R Fadel HM Youssef EM Helmy YA. Role of freshwater fishes in the epidemiology of some zoonotic trematodes in Ismailia province. SCVMJ. (2008) XIII:653–76.

[5] Saad SM Salem AM Mahdy OA Ibrahim ES. Prevalence of metacercarial infection in some marketed fish in Giza governorate, Egypt. J Egypt Soc Parasitol. (2019) 49:129–34. 10.21608/jesp.2019.68295

[6] Khalefa HS Attia MM Abdelsalam M Mahmoud MA Ewiss Z. Immunological status of some edible fishes exposed to parasitic infections in relation to heavy metals pollution. J Parasit Dis. (2022) 1–11. 10.1007/s12639-022-01479-1 PMC9458834 36091276

[7] Mahdy OA Abdel-Maogood SZ Abdelsalam M Shaalan M Abdelrahman HA Salem MA. Epidemiological study of fish-borne zoonotic trematodes infecting nile tilapia with first molecular characterization of two heterophyid flukes. Aquac Res. (2021) 52:4475–88. 10.1111/are.15286

[8] Aly S Eissa I Badran A Elamie M Hussein B. Pathological studies on encysted metacercariae infections among some freshwater fish in Egyptian aquaculture. In: Duetscher Tropentag. Stuttgart: Hohenham University (2005).

[9] Achatz TJ Pulis EE Junker K Binh TT Snyder SD Tkach VV. Molecular phylogeny of the Cyathocotylidae (Digenea, Diplostomoidea) necessitates systematic changes and reveals a history of host and environment switches. Zool Scrip. (2019) 48:545–56. 10.1111/zsc.12360 31937984 PMC6959977

[10] Dzikowski R Levy M Poore M Flowers J Paperna I. Use of rDNA polymorphism for identification of Heterophyidae infecting freshwater fishes. Dis Aquat Organ. (2004) 59:35–41. 10.3354/dao059035 15212290

[11] Locke SA Van Dam A Caffara M Pinto HA López-Hernández D Blanar CA. Validity of the Diplostomoidea and Diplostomida (Digenea, Platyhelminthes) upheld in phylogenomic analysis. Int J Parasitol. (2018) 48:1043–59. 10.1016/j.ijpara.2018.07.001 30347194

[12] Hernández-Mena DI García-Varela M de León GP-P. Filling the gaps in the classification of the Digenea Carus, 1863: systematic position of the Proterodiplostomidae Dubois, 1936 within the superfamily Diplostomoidea Poirier, 1886, inferred from nuclear and mitochondrial DNA sequences. Syst Parasitol. (2017) 94:833–48. 10.1007/s11230-017-9745-1 28822036

[13] El-Bahy NM Bazh EK Sorour SS Elhawary NM. Molecular characterization of the unique *Mesostephanus appendiculatus* (Trematoda: Cyathocotylidae) by small ribosomal RNA from Egypt. Parasitol Res. (2017) 116:1129–36. 10.1007/s00436-016-5342-5 28213655

[14] El-Gayar AK Aly SM. Studies on some protozoan parasites and encysted metacercarial infection of freshwater fishes in Egypt. EVMSPJ. (2013) 9:31–43.

[15] Saoud M Ramadan M. On a new trematode, *Prohemistomum azimi* N. sp. (Trematoda: Cyathocotylidae) from the Egyptian slit-faced bat. Z Parasit. (1977) 53:281–5. 10.1007/BF00389945 595795

[16] Saari S Nareaho A Nikander S. Canine Parasites and Parasitic Diseases. London: Academic press (2018).

[17] Mahfouz ME Mira NM Amer S. Histopathological and molecular studies on the intestine of experimentally infected mice by *Prohemistomum vivax* (Trematoda: Cyathocotylidae). Egypt J Exp Biol. (2005) 1:77–86.

[18] Amer S. Immunological Studies on a Mammalian Host after Infection with Two Trematode Species. (Master Thesis): Tanta (Egypt): Tanta University. (1992).

[19] Shin E-H Kim T-H Hong S-J Park J-H Guk S-M Chai J-Y. Effects of anti-allergic drugs on intestinal mastocytosis and worm expulsion of rats infected with *Neodiplostomum seoulense*. Korean J Parasitol. (2003) 41:81–7. 10.3347/kjp.2003.41.2.81 12815318 PMC2717495

[20] Coakley G Harris NL. Interactions between macrophages and helminths. Parasite Immunol. (2020) 42:e12717. 10.1111/pim.12717 32249432

[21] Maizels RM Yazdanbakhsh M. Immune regulation by helminth parasites: cellular and molecular mechanisms. Nat Rev Immunol. (2003) 3:733–44. 10.1038/nri1183 12949497

[22] Salem HM Khattab MS Yehia N Abd El-Hack ME El-Saadony MT Alhimaidi AR. Morphological and molecular characterization of *Ascaridia columbae* in the domestic pigeon (*Columba livia domestica*) and the assessment of its immunological responses. Poult Sci. (2022) 101:101596. 10.1016/j.psj.2021.101596 34929441 PMC8693010

[23] Olias P Meyer A Klopfleisch R Lierz M Kaspers B Gruber AD. Modulation of the host Th1 immune response in pigeon protozoal encephalitis caused by *Sarcocystis calchasi*. Vet Res. (2013) 44:10. 10.1186/1297-9716-44-10 23398807 PMC3598538

[24] Yasuda K Nakanishi K. Host responses to intestinal nematodes. Int Immunol. (2018) 30:93–102. 10.1093/intimm/dxy002 29346656

[25] Brunet L Joseph S Dunne D Fried B. Immune responses during the acute stages of infection with the intestinal trematode *Echinostoma caproni*. Parasitology. (2000) 120:565–71. 10.1017/S0031182099006009 10874719

[26] Shin E-H Lee S-H Kim J-L Park Y-K Chai J-Y. T-Helper-1 and T-Helper-2 immune responses in mice infected with the intestinal fluke *Neodiplostomum seoulense*: their possible roles in worm expulsion and host fatality. J Parasitol. (2007) 93:1036–45. 10.1645/GE-1203R.1 18163337

[27] Trelis M Sotillo J Monteagudo C Fried B Marcilla A Esteban JG. *Echinostoma caproni* (trematoda): differential *in vivo* cytokine responses in high and low compatible hosts. Exp Parasitol. (2011) 127:387–97. 10.1016/j.exppara.2010.09.004 20849850

[28] Klimpel S Kuhn T Münster J Dörge DD Klapper R Kochmann J. Parasites of Marine Fish and Cephalopods: A Practical Guide. Cham: Springer. (2019). 10.1007/978-3-030-16220-7

[29] Patarwut L Chontananarth T Chai J-Y Purivirojkul W. Infections of digenetic trematode metacercariae in wrestling halfbeak, *Dermogenys pusilla* from Bangkok metropolitan region in Thailand. Korean J Parasitol. (2020) 58:27. 10.3347/kjp.2020.58.1.27 32145724 PMC7066442

[30] Odhner T. Zum natürlichen system der digenen trematoden. Vi Die ableitung der holostomiden und die homologien ihrer haftorgane. Zool Anz. (1913) 42:289–317.

[31] Dubois G. Nouveaux principes de classification des trématodes du groupe des strigeida (note préliminaire). Rev Suisse Zool. (1936) 43:507–15. 10.5962/bhl.part.117684

[32] Fahmy M Selim M. Studies on some trematode parasites of dogs in Egypt with special reference to the role played by fish in their transmission. Z Parasitenkd. (1959) 19:3–13. 10.1007/BF00260314 13660200

[33] El-Naffar M Saoud M Hassan I. Role played by fish in transmitting some trematodes of dogs and cats at Aswan province A. R. Egypt. Assiut Vet Med J. (1985) 14:57–67.

[34] Alghabban AJM. Fish farms as a source for parasites transport: parasitological and developmental studies of *Prohemistomum vivax* with the ameliorating role of *Moringa oleifera* in the treatment. J Am sci. (2014) 10:6–14.

[35] Bowles J McManus DP. Rapid discrimination of *Echinococcus* species and strains using a polymerase chain reaction-based RFLP method. Mol Biochem Parasitol. (1993) 57:231–9. 10.1016/0166-6851(93)90199-8 8094539

[36] Bowles J Blair D McManus D. A molecular phylogeny of the human schistosomes. Mol Phylogenet Evol. (1995) 4:103–9. 10.1006/mpev.1995.1011 7663756

[37] Kumar S Stecher G Li M Knyaz C Tamura K. Mega X: molecular evolutionary genetics analysis across computing platforms. Mol Biol Evol. (2018) 35:1547. 10.1093/molbev/msy096 29722887 PMC5967553

[38] Larkin MA Blackshields G Brown NP Chenna R McGettigan PA McWilliam H. Clustal W and clustal X version 2.0. Bioinformatics. (2007) 23:2947–8. 10.1093/bioinformatics/btm404 17846036

[39] Zhang Y Zhao X Zhu Y Ma J Ma H Zhang H. Probiotic mixture protects dextran sulfate sodium-induced colitis by altering tight junction protein expressions and increasing tregs. Mediators Inflamm. (2018) 2018:9416391. 10.1155/2018/9416391 29849501 PMC5925202

[40] Livak KJ Schmittgen TD. Analysis of relative gene expression data using real-time quantitative PCR and the 2^−Δ*ΔCT*^ method. Methods. (2001) 25:402–8. 10.1006/meth.2001.1262 11846609

[41] Hayashi T Hiromoto Y Chaichoune K Patchimasiri T Chakritbudsabong W Prayoonwong N. Host cytokine responses of pigeons infected with highly pathogenic Thai avian influenza viruses of subtype H5N1 isolated from wild birds. PLoS ONE. (2011) 6:e23103. 10.1371/journal.pone.0023103 21826229 PMC3149639

[42] Fahmy M Arafa M Khalifa R Abdel-Rahman A Mounib M. Studies on helminth parasites in some small mammals in Assuit governorate. 1. Trematode parasites. Assiut Vet Med J. (1984) 11:43–52. 10.21608/avmj.1984.190819

[43] Ashour DS Othman AA Radi DA. Insights into regulatory molecules of intestinal epithelial cell turnover during experimental infection by *Heterophyes heterophyes*. Exp Parasitol. (2014) 143:48–54. 10.1016/j.exppara.2014.05.003 24852217

[44] Songsri J Aukkanimart R Boonmars T Ratanasuwan P Laummaunwai P Sriraj P. Animal models for *Echinostoma malayanum* infection: worm recovery and some pathology. Korean J Parasitol. (2016) 54:47–53. 10.3347/kjp.2016.54.1.47 26951978 PMC4792317

[45] Khalil A Helal I. Scanning electron microscopy of the tegumental surface of adult *Prohemistomum vivax* (Sonsino, 1892) Azim, 1933 (Trematoda: Cythocotylidae). J Egypt Germ Soc Zool. (1992) 7:459–73.

[46] Chai J-Y Lee H-S Hong S-J Yoo JH Guk S-M Seo M. Intestinal histopathology and *in situ* postures of *Gymnophalloides seoi* in experimentally infected mice. Korean J Parasitol. (2001) 39:31. 10.3347/kjp.2001.39.1.31 11301588 PMC2721063

[47] Chai J-Y. Study on Metagonimus yokogawai (Katsurada, 1912) in Korea V. Intestinal Pathology in Experimentally Infected Albino Rats. Seoul National University, College of Medicine (1979) 20:104–117.

[48] Helal IB Agamy EEDI Amer SE. Interaction between *Toxocara vitulorum* [Nematoda] and *Prohemistomum vivax* [Trematoda]. J Med Res Instit. (1998) 19:166–82.

[49] Little MC Bell LV Cliffe LJ Else KJ. The characterization of intraepithelial lymphocytes, lamina propria leukocytes, and isolated lymphoid follicles in the large intestine of mice infected with the intestinal nematode parasite *Trichuris muris*. J Immunol. (2005) 175:6713–22. 10.4049/jimmunol.175.10.6713 16272327

[50] Shi W Wei ZY Elsheikha HM Zhang FK Sheng ZA Lu KJ. Dynamic expression of cytokine and transcription factor genes during experimental *Fasciola gigantica* infection in buffaloes. Parasit Vect. (2017) 10:602. 10.1186/s13071-017-2538-1 29216911 PMC5721666

[51] Zhang B Jiao Y Cai W Tao J Liu R. Influence of interferon gamma treatment on expression of TGF-beta1 and its receptors in liver fibrosis of mice with schistosomiasis japonica. Zhongguo Ji Sheng Chong Xue Yu Ji Sheng Chong Bing Za Zhi. (2004) 22:340–3. 15830858

[52] Mondragón-De-La-Pena C Ramos-Solis S Barbosa-Cisneros O Rodriguez-Padilla C Tavizon-Garcia P Herrera-Esparza R. *Echinococcus granulosus* down regulates the hepatic expression of inflammatory cytokines IL-6 and TNFα in BALB/c mice. Parasite. (2002) 9:351–6. 10.1051/parasite/2002094351 12514950

[53] Su Z Segura M Morgan K Loredo-Osti JC Stevenson MM. Impairment of protective immunity to blood-stage malaria by concurrent nematode infection. Infect Immun. (2005) 73:3531–9. 10.1128/IAI.73.6.3531-3539.2005 15908382 PMC1111846

[54] Roncarolo MG Bacchetta R Bordignon C Narula S Levings MK. Type 1 T regulatory cells. Immunol Rev. (2001) 182:68–79. 10.1034/j.1600-065X.2001.1820105.x 11722624

[55] O'Shea JJ Gadina M Siegel RM. 9—cytokines and Cytokine receptors. In: Rich RR, Fleisher TA, Shearer WT, Schroeder HW, Frew AJ, Weyand CM, editors. Clinical Immunology. 5th edition. London: Elsevier (2019). p. 127–55.e1.

[56] Abbas AK Trotta E Simeonov RD Marson A Bluestone JA. Revisiting IL-2: biology and therapeutic prospects. Sci Immunol. (2018) 3:eaat1482. 10.1126/sciimmunol.aat1482 29980618

[57] Hodge DL Martinez A Julias JG Taylor LS Young HA. Regulation of nuclear gamma interferon gene expression by interleukin 12 (IL-12) and IL-2 represents a novel form of posttranscriptional control. Mol Cell Biol. (2002) 22:1742–53. 10.1128/MCB.22.6.1742-1753.2002 11865054 PMC135596

[58] Burke JD Young HA. IFN-γ: a cytokine at the right time, is in the right place. Semin Immunol. (2019) 43:101280. 10.1016/j.smim.2019.05.002 31221552 PMC7367502

[59] Yasuda K Kuroda E. Role of eosinophils in protective immunity against secondary nematode infections. Immunol Med. (2019) 42:148–55. 10.1080/25785826.2019.1697135 31794348

[60] Zhang Y Derycke L Holtappels G Wang X Zhang L Bachert C. Th2 cytokines orchestrate the secretion of MUC5AC and MUC5B in IL-5-positive chronic rhinosinusitis with nasal polyps. Allergy. (2019) 74:131–40. 10.1111/all.13489 29802623

[61] Ruiz-Manzano RA Hernández-Cervantes R Del Río-Araiza VH Palacios-Arreola MI Nava-Castro KE Morales-Montor J. Immune response to chronic *Toxocara canis* infection in a mice model. Parasite Immunol. (2019) 41:e12672. 10.1111/pim.12672 31557337

[62] Li L Si H Wu S-W Mendez JO Zarlenga D Tuo W. Characterization of IL-10-producing neutrophils in cattle infected with *Ostertagia ostertagi*. Sci Rep. (2019) 9:20292. 10.1038/s41598-019-56824-x 31889109 PMC6937330

[63] Rodrigues VF Bahia MPS Cândido NR Moreira JMP Oliveira VG Araújo ES. Acute infection with *Strongyloides venezuelensis* increases intestine production IL-10, reduces Th1/Th2/Th17 induction in colon and attenuates dextran sulfate sodium-induced colitis in BALB/c mice. Cytokine. (2018) 111:72–83. 10.1016/j.cyto.2018.08.003 30118915

[64] Farid AS Fath EM Mido S Nonaka N Horii Y. Hepatoprotective immune response during *Trichinella spiralis* infection in mice. J Vet Med Sci. (2018) 81:169–76. 10.1292/jvms.18-0540 30541982 PMC6395222

[65] Liu Q Liu Z Rozo CT Hamed HA Alem F Urban JF. The role of B cells in the development of CD4 effector T cells during a polarized Th2 immune response. J Immunol. (2007) 179:3821–30. 10.4049/jimmunol.179.6.3821 17785819 PMC2258088

[66] Iwasaki A Kelsall BL. Freshly isolated peyer's patch, but not spleen, dendritic cells produce interleukin 10 and induce the differentiation of T helper type 2 cells. J Exp Med. (1999) 190:229–40. 10.1084/jem.190.2.229 10432286 PMC2195574

[67] Alhallaf R Agha Z Miller CM Robertson AA Sotillo J Croese J. The NLRP3 inflammasome suppresses protective immunity to gastrointestinal helminth infection. Cell Rep. (2018) 23:1085–98. 10.1016/j.celrep.2018.03.097 29694887

[68] Sims JE Smith DE. The IL-1 family: regulators of immunity. Nat Rev Immunol. (2010) 10:89–102. 10.1038/nri2691 20081871

[69] Helmby H Grencis RK. Interleukin 1 plays a major role in the development of Th2-mediated immunity. Eur J Immunol. (2004) 34:3674–81. 10.1002/eji.200425452 15549727

[70] O'Neill LA. The interleukin-1 receptor/toll-like receptor superfamily: 10 years of progress. Immunol Rev. (2008) 226:10–8. 10.1111/j.1600-065X.2008.00701.x 19161412

[71] Shibuya K Robinson D Zonin F Hartley SB Macatonia SE Somoza C. IL-1α and TNF- α are required for IL-12-induced development of Th1 cells producing high levels of IFN-γ in BALB/c but not C57Bl/6 mice. J Immunol. (1998) 160:1708–16. 9469428

[72] Neurath MF Finotto S. IL-6 signaling in autoimmunity, chronic inflammation and inflammation-associated cancer. Cytokine Growth Factor Rev. (2011) 22:83–9. 10.1016/j.cytogfr.2011.02.003 21377916

[73] Febbraio MA Pedersen BK. Contraction-induced myokine production and release: is skeletal muscle an endocrine organ? Exerc Sport Sci Rev. (2005) 33:114–9. 10.1097/00003677-200507000-00003 16006818

[74] Secombes CJ Wang T Bird S. Chapter 5 - Vertebrate cytokines and their evolution. In: Malagoli D, editor. The Evolution of the Immune System. London: Academic Press (2016). p. 87–150. 10.1016/B978-0-12-801975-7.00005-0

[75] Zhang S Zhao J Bai X Handley M Shan F. Biological effects of IL-15 on immune cells and its potential for the treatment of cancer. Int Immunopharmacol. (2021) 91:107318. 10.1016/j.intimp.2020.107318 33383444

[76] Yang Y Lundqvist A. Immunomodulatory effects of IL-2 and IL-15; implications for cancer immunotherapy. Cancers. (2020) 12:3586. 10.3390/cancers12123586 33266177 PMC7761238

[77] Milano S Di Bella G D'Agostino P Barbera C Caruso R La Rosa M. IL-15 in human visceral leishmaniasis caused by *Leishmania infantum*. Clin Exp Immunol. (2002) 127:360–5. 10.1046/j.1365-2249.2002.01749.x 11876762 PMC1906348

[78] Silva LLdL Gomes RS Silva MVT Joosten LAB Ribeiro-Dias F. IL-15 enhances the capacity of primary human macrophages to control *Leishmania braziliensis* infection by IL-32/vitamin D dependent and independent pathways. Parasitol Int. (2020) 76:102097. 10.1016/j.parint.2020.102097 32114085

[79] Burrack KS Huggins MA Taras E Dougherty P Henzler CM Yang R. Interleukin-15 complex treatment protects mice from cerebral malaria by inducing interleukin-10-producing natural killer cells. Immunity. (2018) 48:760–72.e4. 10.1016/j.immuni.2018.03.012 29625893 PMC5906161

[80] Dann SM Wang H-C Gambarin KJ Actor JK Robinson P Lewis DE. Interleukin-15 activates human natural killer cells to clear the intestinal protozoan *Cryptosporidium*. J Infect Dis. (2005) 192:1294–302. 10.1086/444393 16136475

[81] Bhadra R Guan H Khan IA. Absence of both IL-7 and IL-15 severely impairs the development of CD8+ T cell response against *Toxoplasma gondii*. PLoS ONE. (2010) 5:e10842. 10.1371/journal.pone.0010842 20520779 PMC2877110

[82] Almeria S Canals A Gómez-Muñoz MT Zarlenga DS Gasbarre LC. Characterization of protective immune responses in local lymphoid tissues after drug-attenuated infections with *Ostertagia ostertagi* in calves. Vet Parasitol. (1998) 80:53–64. 10.1016/S0304-4017(98)00185-X 9877071

[83] Abdel-Latif M Abdel-Aziz AM Taha R El-Mallah A-M Sami S. Filarial excretory-secretory antigens induced murine production of IFN-gamma and IL-15. EJZ. (2016) 65:65–88. 10.12816/0027818

[84] Mishra P Palma M Bleich D Loke P Gause W. Systemic impact of intestinal helminth infections. Mucosal Immunol. (2014) 7:753–62. 10.1038/mi.2014.23 24736234

